# Teplizumab induces persistent changes in the antigen-specific repertoire in individuals at risk for type 1 diabetes

**DOI:** 10.1172/JCI177492

**Published:** 2024-08-13

**Authors:** Ana Lledó-Delgado, Paula Preston-Hurlburt, Sophia Currie, Pamela Clark, Peter S. Linsley, S. Alice Long, Can Liu, Galina Koroleva, Andrew J. Martins, John S. Tsang, Kevan C. Herold

**Affiliations:** 1 Departments of Immunobiology and Internal Medicine, Yale University School of Medicine, New Haven, Connecticut, USA.; 2 Benaroya Research Institute, Seattle, Washington, USA.; 3 Center for Systems and Engineering Immunology and Department of Immunobiology, Yale University School of Medicine, New Haven, Connecticut, USA.; 4 NIH Center for Human Immunology, National Institutes of Health, Bethesda, Maryland, USA.; 5 Department of Biomedical Engineering, Yale University, New Haven, Connecticut, USA.

**Keywords:** Autoimmunity, Endocrinology, Adaptive immunity, T cells, Tolerance

## Abstract

**BACKGROUND:**

Teplizumab, a non-FcR-binding anti-CD3 mAb, is approved to delay progression of type 1 diabetes (T1D) in at-risk patients. Previous investigations described the immediate effects of the 14-day treatment, but longer-term effects of the drug remain unknown.

**METHODS:**

With an extended analysis of study participants, we found that 36% were undiagnosed or remained free of clinical diabetes after 5 years, suggesting operational tolerance. Using single-cell RNA sequencing, we compared the phenotypes, transcriptome, and repertoire of peripheral blood CD8^+^ T cells including autoreactive T cells from study participants before and after teplizumab and features of responders and non-responders.

**RESULTS:**

At 3 months, there were transcriptional signatures of cell activation in CD4^+^ and CD8^+^ T cells including signaling that was reversed at 18 months. At that time, there was reduced expression of genes in T cell receptor and activation pathways in clinical responders. In CD8^+^ T cells, we found increased expression of genes associated with exhaustion and immune regulation with teplizumab treatment. These transcriptional features were further confirmed in an independent cohort. Pseudotime analysis showed differentiation of CD8^+^ exhausted and memory cells with teplizumab treatment. *IL7R* expression was reduced, and patients with lower expression of CD127 had longer diabetes-free intervals. In addition, the frequency of autoantigen-reactive CD8^+^ T cells, which expanded in the placebo group over 18 months, did not increase in the teplizumab group.

**CONCLUSION:**

These findings indicate that teplizumab promotes operational tolerance in T1D, involving activation followed by exhaustion and regulation, and prevents expansion of autoreactive T cells.

**TRIAL REGISTRATION:**

ClinicalTrials.gov NCT01030861.

**FUNDING:**

National Institute of Diabetes and Digestive and Kidney Diseases/NIH, Juvenile Diabetes Research Foundation.

## Abstract 2

We show, in the anti-CD3 mAb (teplizumab) prevention trial that a single course of teplizumab causes changes in immune cells that persist for 18 months after treatment. Treatment prevents the expansion of autoantigen specific CD8+ T cells

## Graphical abstract

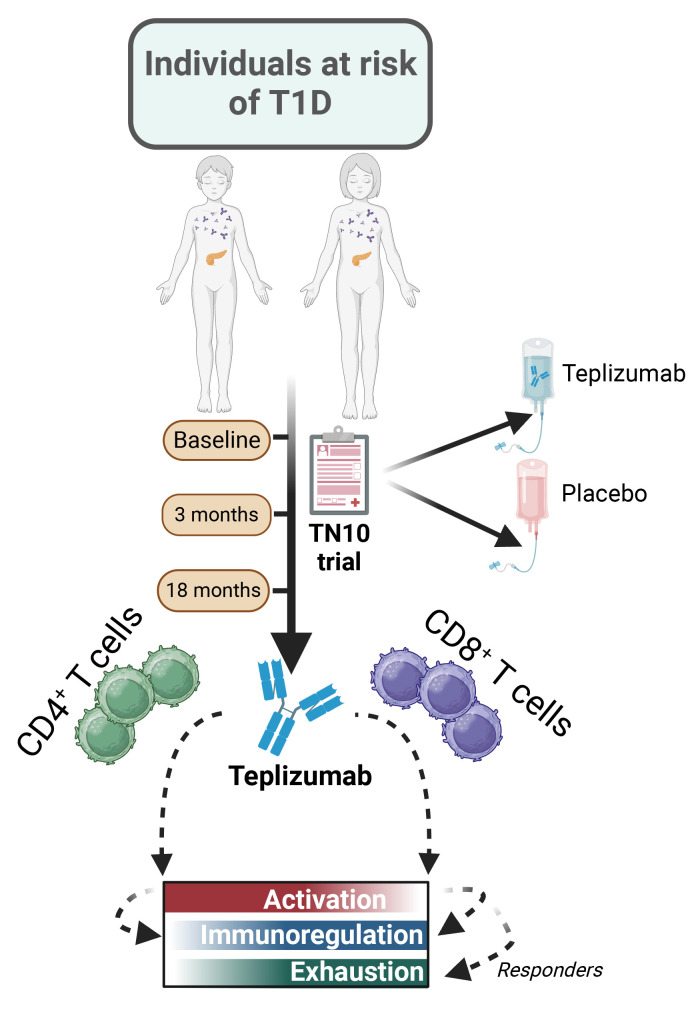

## Introduction

The TrialNet Teplizumab Prevention Study (TN10) was a randomized placebo-controlled trial to test whether a single 14-day course of treatment with teplizumab, a non-FcR-binding anti-CD3 mAb, would delay or prevent the clinical diagnosis of type 1 diabetes (T1D) (i.e., stage 3 T1D) in individuals at high risk (i.e., with stage 2 T1D). The single 14-day course of teplizumab caused a significant delay in progression to the diagnosis (i.e., stage 3 T1D) (1). Prior investigations showed that teplizumab treatment induced a signature of partial exhaustion in CD8^+^ T cells (2, 3). The phenotypic changes that identified these cells, KLRG1 and TIGIT, peaked 6 months after a 14-day course of treatment but returned to baseline levels by 18 months (2, 3). Despite the absence of mAb in the circulation or on the surfaces of cells, and a decline in the frequency of the KLRG1^+^TIGIT^+^ CD8^+^ T cells after treatment, there were extended effects on autoimmunity (1–5). In our previous reports, the median time to diagnosis with stage 3 T1D was 59.6 months, nearly 5 years after the 14-day course of drug. This outcome suggested the induction of operational tolerance since the immune therapy was not given continuously. Understanding the mechanisms whereby teplizumab has lasting effects on immune responses and the reasons why some patients but not others have persistent responses is important for explaining the basis of human immune tolerance, selecting patients to treat, and guiding decisions regarding the ideal time for retreatment or the potential need for combination therapies.

Studies in NOD mice indicated that non-FcR-binding anti-CD3 mAb induced a selective depletion of effector T cells located in the islets of Langerhans (6). Depletion of T cells alone is unlikely to completely account for the drug mechanisms of action since, in humans, the rate of recovery of T cells into the peripheral circulation following their decline is unlikely to be explained by the development of new cells or emergence of thymic emigrants (7). In NOD mice and humanized mice, anti-CD3 mAbs trigger the egress of peripheral blood T cells to the gut wall, which may account for the rapid decline of cells in the peripheral blood in patients (8). Furthermore, in mice, there was induction of an inflammatory signal, with production of IL-17a, in the gut wall, where the environment fostered the development of T cells with regulatory phenotypes with characteristics such as expression of TGF-β and IL-10 (9). In addition to these actions, the effects on CD4^+^ cells, needed for disease development, have not been analyzed. Moreover, although there are broad changes in T cells with treatment, the effects of teplizumab on the autoantigen-reactive repertoire, specifically on the CD8^+^ T cells that are thought to cause disease, in mice or humans are not known. For example, it is not clear whether depletion of these cells occurs or contributes to arresting disease progression.

To understand the effects of teplizumab treatment in humans and to identify cellular signatures that may be associated with the long responses after a single course of the drug, we analyzed peripheral blood cells from participants in the TN10 trial with stage 2 T1D (i.e., 2 positive autoantibodies and dysglycemia) who were at high risk for progression to clinical stage 3 T1D. We identified changes in circulating peripheral blood mononuclear cells (PBMCs) using flow cytometry, single-cell RNA sequencing (scRNA-Seq), and bulk RNA-Seq to analyze the transcriptome and phenotypes of cells before and up to 18 months after treatment with teplizumab or placebo. We differentiated the effects of drug treatment on patients who showed poor and robust clinical responses to drug treatment. There was initially an increase in transcriptomic signatures of cell activation of CD8^+^ T cells followed by a decline and differentiation of cells into effector cells with features of exhaustion and regulation at 18 months. Similar changes in CD4^+^ T cells occurred, with activation initially and a decline in the responders at 18 months. Teplizumab treatment reduced expression of *IL7R* in CD8^+^ T cells, which is needed for their growth and expansion. Consistent with this finding, there was reduced expansion of autoantigen-reactive CD8^+^ T cells in teplizumab-treated patients compared with those treated with placebo. These observations suggest that the signals delivered by the anti-CD3 mAb affected multiple immune cell subsets, including differentiation into CD8^+^ cells with regulatory and exhaustion features and prevention of expansion of autoantigen-specific CD8^+^ T cells. These mechanisms may account for the induction of operational tolerance with a single course of teplizumab treatment.

## Results

### Persistence of clinical responses to teplizumab and changes in immune cell subsets.

**Figure 1:**
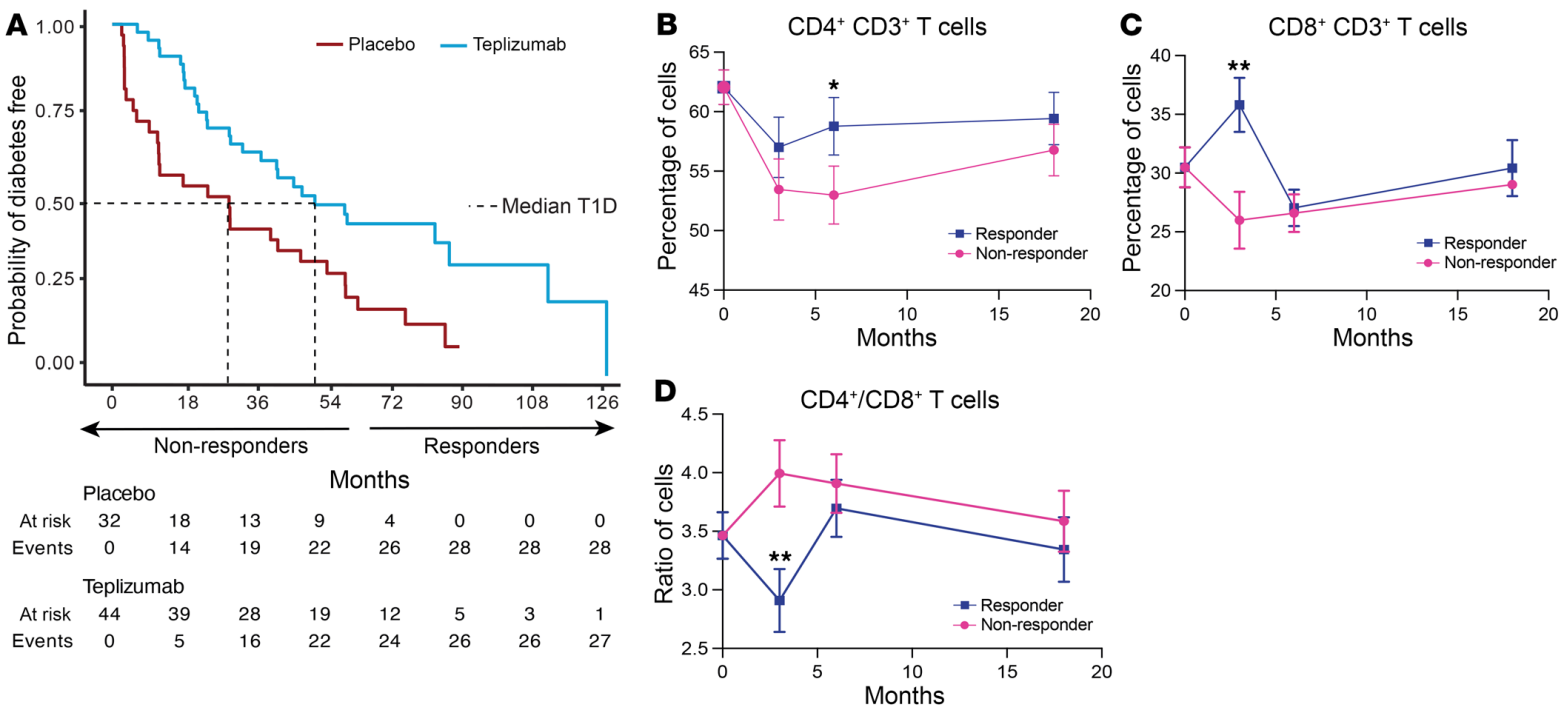
Persistence of clinical responses to teplizumab. (**A**) Kaplan-Meier curve showing the progression from stage 2 T1D to stage 3 T1D in TN10 study participants who were treated with teplizumab (*n* = 44) or placebo (*n* = 32). Median times to development of stage 3 T1D: teplizumab, 52.2 months (95% CI: 30.5–86.9); placebo, 27.3 months (95% CI: 9.5–48.4) (log rank *P* = 0.0026). (**B**–**D**) Percentage of CD4^+^ T cells (**B**) and CD8^+^ T cells (**C**) and ratio of CD4^+^ to CD8^+^ T cells (**D**) by flow cytometry in clinical responders (*n* = 17) and non-responders (*n* = 27) to teplizumab treatment (*P < 0.05, **P < 0.01 by repeated-measures ANOVA corrected for the baseline values). Mean ± SEM from the mixed model is shown.

We followed the 76 participants with stage 2 T1D (i.e., ≥2 positive autoantibodies with dysglycemia) who had enrolled in the TN10 anti-CD3 mAb (teplizumab) trial to determine whether a single 14-day course of teplizumab treatment would delay progression to the clinical diagnosis of stage 3 T1D. The median follow-up time was 80.46 months (Supplemental Figure 1; supplemental material available online with this article; https://doi.org/10.1172/JCI177492DS1). The median time to diagnosis with clinical stage 3 T1D was 52.2 (95% CI: 30.5–86.7) months in the teplizumab and 27.3 (95% CI: 9.5–48.4) months in the placebo group (*P* = 0.0026, log-rank test) (Figure 1A). At the end of the follow-up, 16 of 44 (36%) had not been diagnosed with stage 3 T1D versus 4 of 32 (12.5%) in the placebo group (*P* = 0.03 Fisher’s exact test). Among the teplizumab-treated participants, 17 were classified as responders because they remained undiagnosed or were diagnosed with stage 3 after 60 months. The other 27 participants were classified as non-responders.

We analyzed the changes in CD4^+^ and CD8^+^ T cells after treatment with placebo or teplizumab (*n* = 65) and among the teplizumab-treated patients (*n* = 38) who were classified as responders or non-responders by flow cytometry. The CD3^+^, CD4^+^, and CD8^+^ T cell counts did not change significantly in either treatment arm (Supplemental Figure 2A). However, within the CD3 compartment, we found statistically significant differences in the relative frequency of the cells in teplizumab-treated responders and non-responders. In the responders, there was an increase in the percentage of CD4^+^ cells at 6 months (*P* < 0.05) (Figure 1B) and in the CD8^+^ cells at 3 months (*P* < 0.01) (Figure 1C). The ratio of CD4^+^ to CD8^+^ cells was decreased at 3 months in the responders (*P* < 0.01) (Figure 1D).

### Cellular signatures and responses 3 months and 18 months after treatment with teplizumab.

To determine changes among cell subsets, we analyzed by scRNA-Seq the transcriptomes of cell subsets from teplizumab- and placebo-treated patients collected at the baseline (*n* = 7 teplizumab, *n* = 7 placebo) (Supplemental Figure 2B), 3 months (*n* = 7 teplizumab, *n* = 7 placebo) (Supplemental Figure 2C), and 18 months (*n* = 7 teplizumab, *n* = 6 placebo) (Supplemental Figure 2D). In our analysis, we excluded genes that were significantly different between the study arms before treatment. We annotated 5 major cell clusters based on the expression of key identity genes: CD8^+^ T cells (*CD8A*^hi^, *CD3D*^hi^, *CD3G*^hi^), CD4^+^ T cells (*CD8A*^–^, *CD3D*^hi^, *CD3G*^hi^), B cells (*CD79A*^hi^, *MS4A1*^hi^), monocytes (*CD14*^hi^, *FCGR3A*^hi^), and dendritic cells (*PLD4*^hi^, *LILRA4*^hi^) (Supplemental Figure 2E and Supplemental Figure 3).

**Figure 2:**
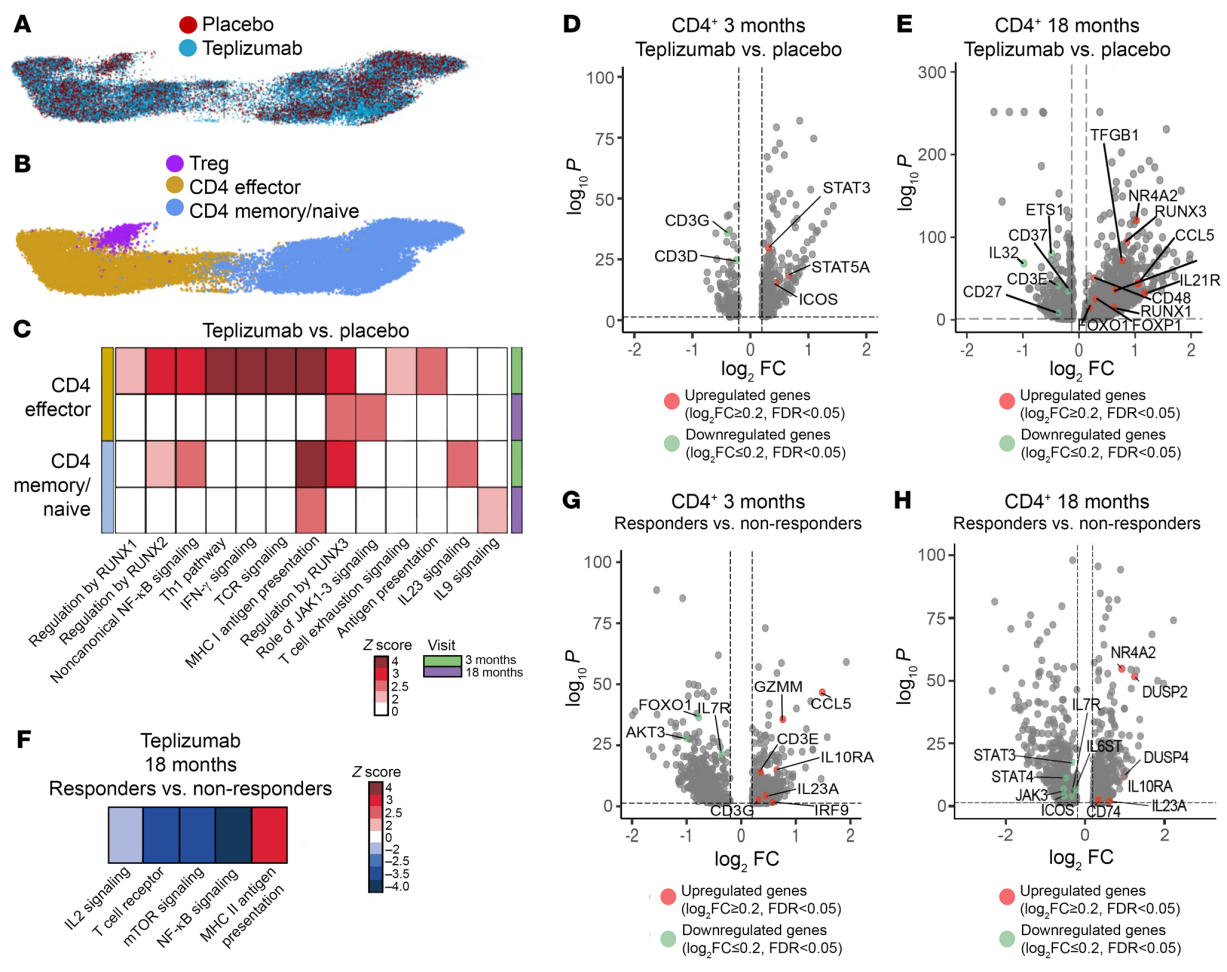
Changes across time in CD4^+^ T cells after teplizumab treatment. (**A** and **B**) 3D UMAP visualization of CD4^+^ cells at 18 months. Points represent individual cells, and colors denote treatment arm (**A**) and clusters (**B**) as labeled (*n* = 7 teplizumab, *n* = 6 placebo). (**C**) Heatmap showing the *z* score of the pathways with statistically significant differences in the CD4^+^ T cell clusters at 3-month (*n* = 7 teplizumab, *n* = 7 placebo) and 18-month visit (*n* = 7 teplizumab, *n* = 6 placebo). The pathways were inferred based on the differentially expressed genes (DEGs) between teplizumab and placebo groups and selected based on adjusted *P* < 0.05 by IPA software. Blue scale denotes grades of prediction of lower enrichment, while red scale indicates higher enrichment of the pathways based on the *z* score. (**D** and **E**) Volcano plot visualization of the DEGs in teplizumab versus placebo in the CD4^+^ T cells at 3 months (**D**) and 18 months (**E**). The dashed lines identify average log_2_ fold change (log_2_FC) ≥ |0.2| and adjusted *P* value < 0.05. (**F**) Heatmap showing the *z* score of the pathways with statistically significant differences in the CD4^+^ T cell clusters at 18 months in the teplizumab group (*n* = 3 responders, *n* = 4 non-responders). The pathways were inferred based on the DEGs between responder and non-responder individuals and selected based on adjusted *P* < 0.05 by IPA software. Blue scale denotes grades of prediction of lower enrichment, while red scale indicates higher enrichment of the pathways based on the *z* score. (**G** and **H**) Volcano plot visualization of the DEGs in responders versus non-responders in the CD4^+^ T cells at 3 months (**G**) and 18 months (**H**). The dashed lines identify average log_2_FC ≥ |0.2| and adjusted *P* value < 0.05.

In the CD4^+^ T cells, after removal of baseline differences, there were 408 and 1,566 differentially expressed genes (DEGs) (Supplemental Table 1) at 3 and 18 months, respectively. We subdivided the CD4^+^ cells and analyzed the differences between teplizumab and placebo in the subsets differentiating CD4^+^ effector from CD4^+^ memory and naive T cells that were maintained as a cluster owing to the limitation in the number of cells (Figure 2, A and B). There was enrichment of pathways related to antigen presentation, immune regulation, non-canonical NF-κB and IFN-γ signaling, and T cell exhaustion in both effector and memory CD4^+^ T cells at both time points (Figure 2C). The frequency of DEGs among the annotated Tregs was insufficient to infer differences in pathways. At 3 months, there was increased expression of genes involved in T cell stimulation, e.g., *STAT5A*, *STAT3*, and *ICOS* (Figure 2D). At 18 months, these pathways were no longer enriched in the teplizumab-treated patients, but JAK1–3 signaling was enriched in effectors (Figure 2C). In addition, at 18 months, key genes involved in immune regulation such as *TGFB1*, *NR4A2*, *RUNX3*, and *RUNX1* were increased in expression (Figure 2E). There was decreased expression of genes associated with T cell receptor (TCR) signaling, *CD3G* and *CD3E*, suggesting reduced T cell activation at 18 months (Figure 2E).

We also compared the transcriptomes of CD4^+^ T cells, at 3 and 18 months, between the long-term responders and non-responders, excluding the genes that differed at the baseline. There were 891 and 1,049 DEGs between these patients at 3 and 18 months, respectively (Supplemental Table 2). We found downregulation of *IL7R* in responders at 3 months and upregulation of *CD3G* and *CD3E* (Figure 2G), whereas at 18 months, expression of IL10RA and *GATA3* were increased and *IL7R*, *IL6ST*, *ICOS*, and *STAT3* were decreased (Figure 2H). At 18 months, we found reduced enrichment in TCR, mTOR, and NF-κB signaling pathways, whereas the MHC II antigen presentation pathway was enriched in responders(Figure 2F). The evolution of these changes suggests initial activation followed by reduced signaling pathways in the responders.

**Figure 3:**
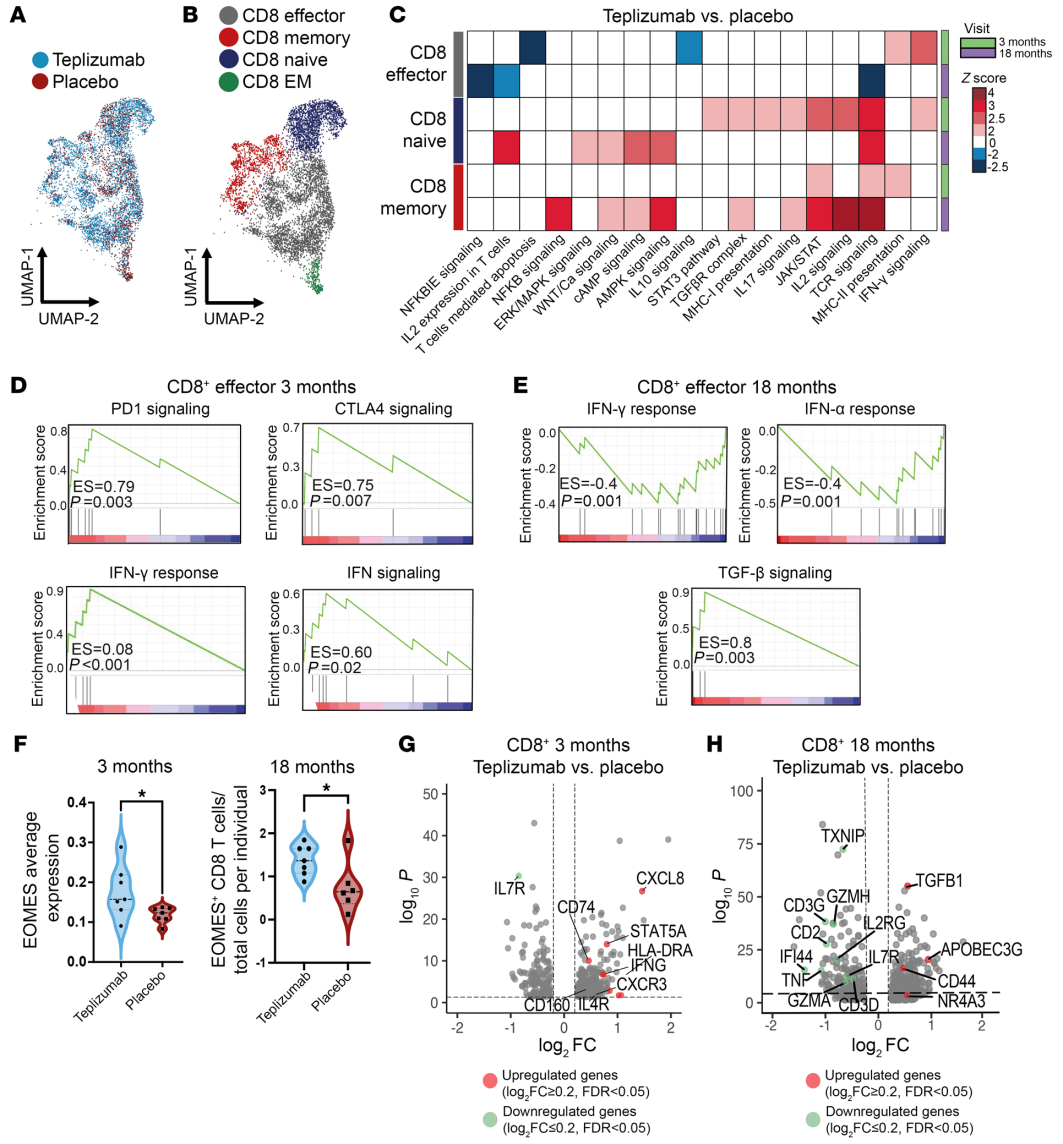
Changes across time in CD8^+^ T cells after teplizumab treatment. (**A** and **B**) 2D UMAP visualization of CD8^+^ cells at 18 months. Points represent individual cells, and colors denote treatment arm (**A**) and clusters (**B**) as labeled (*n* = 7 teplizumab, *n* = 6 placebo). EM, effector memory. (**C**) Heatmap showing *z* score of pathways with statistically significant differences in the CD8^+^ T cell clusters at 3 months (*n* = 7 teplizumab, *n* = 7 placebo) and 18 months (*n* = 7 teplizumab, *n* = 6 placebo). The pathways were inferred based on the DEGs between teplizumab and placebo groups and selected based on adjusted *P* < 0.05 by IPA software. Blue scale denotes grades of prediction of lower enrichment, while red scale indicates higher enrichment of pathways based on the *z* score. (**D**) GSEA of the total CD8^+^ effector T cell cluster in teplizumab versus placebo DEGs at 3 months with enrichment in genes belonging to PD-1 signaling (enrichment score [ES] = 0.79, *P* = 0.003), CTLA4 signaling (ES = 0.75, *P* = 0.007), IFN-γ response (ES = 0.08, *P* < 0.001), and IFN signaling (ES = 0.60, *P* = 0.02). (**E**) GSEA of total CD8^+^ T cell cluster in teplizumab versus placebo DEGs at 18 months with less enrichment in genes belonging to IFN-γ response (ES = –0.4, *P* = 0.001) and INF-α response (ES = –0.48, *P* = 0.01) and higher enrichment in TGF-β signaling (ES = 0.8, *P* = 0.003). (**F**) Violin plot representing average expression of *EOMES* in CD8^+^ T cells at 3 months in teplizumab (*n* = 7) and placebo (*n* = 7) (*P* < 0.05) and the number of CD8^+^ T cells expressing *EOMES* at 18 months in teplizumab (*n* = 7) and placebo (*n* = 6) (*P* < 0.05). (**G** and **H**) Volcano plot visualization of DEGs in teplizumab versus placebo in CD8^+^ T cells at 3 months (*n* = 7 teplizumab, *n* = 7 placebo) (**G**) and 18 months (*n* = 7 teplizumab, *n* = 6 placebo) (**H**). The dashed lines identify average log_2_FC ≥ |0.2| and adjusted *P* value < 0.05.

We categorized CD8^+^ T cells into subclusters of memory, effector, and naive subsets (Figure 3, A and B). After filtering of the baseline differences, there were 412 and 702 DEGs in the total CD8^+^ T cells at 3 and 18 months, respectively (Supplemental Table 1). Using Ingenuity Pathway Analysis (IPA), we found variations in pathway enrichment among the subsets at both time points (Figure 3C). Teplizumab treatment was associated with the enrichment of IFN-γ signaling in effector and naive cells and enhanced antigen presentation pathway within effector and memory CD8^+^ T cells. By 18 months, there was predicted decrease in TCR signaling and IL-2 expression in CD8^+^ effector T cells, while naive and memory CD8^+^ T cells continued to exhibit enriched pathway activity. Additionally, NF-κB signaling was enriched at 18 months in the memory CD8^+^ cells. By gene set enrichment analysis (GSEA), we detected an enrichment of pathways indicative of exhaustion, such as PD-1 signaling and CTLA4 signaling, but also of IFN-γ signaling at 3 months (Figure 3D) in the CD8^+^ effector T cells, whereas these pathways (IFN-γ and IFN-α response) were reduced and TGF-β signaling was increased at 18 months (Figure 3E). *EOMES* expression was increased in the teplizumab group in CD8^+^ T cells at 3 months, and the number of *EOMES*^+^ CD8^+^ T cells was higher at 18 months in the teplizumab group (Figure 3F).

Consistent with the pathway analysis, there was increased expression of genes related to activation (*IFNG*), cytokine activity (*CXCR3*, *CXCL8*), and downregulation of *IL7R* at 3 months (Figure 3G). At 18 months, there was a statistically significant reduction in the expression of cytotoxicity-associated genes (e.g., *GZMA*, *GZMH*, *ISG15*, *IL2RG*, *TNF*) with an increase in others associated with CD8^+^ T cell activation (*CD44*, *APOBEC3G*) (10–12), regulation (e.g., *T*GFβ1), and cell death (*PDCD5*) (13) but a decrease in genes associated with expansion (*IL7R*, *IL2RG*) (Figure 3H).

**Figure 4:**
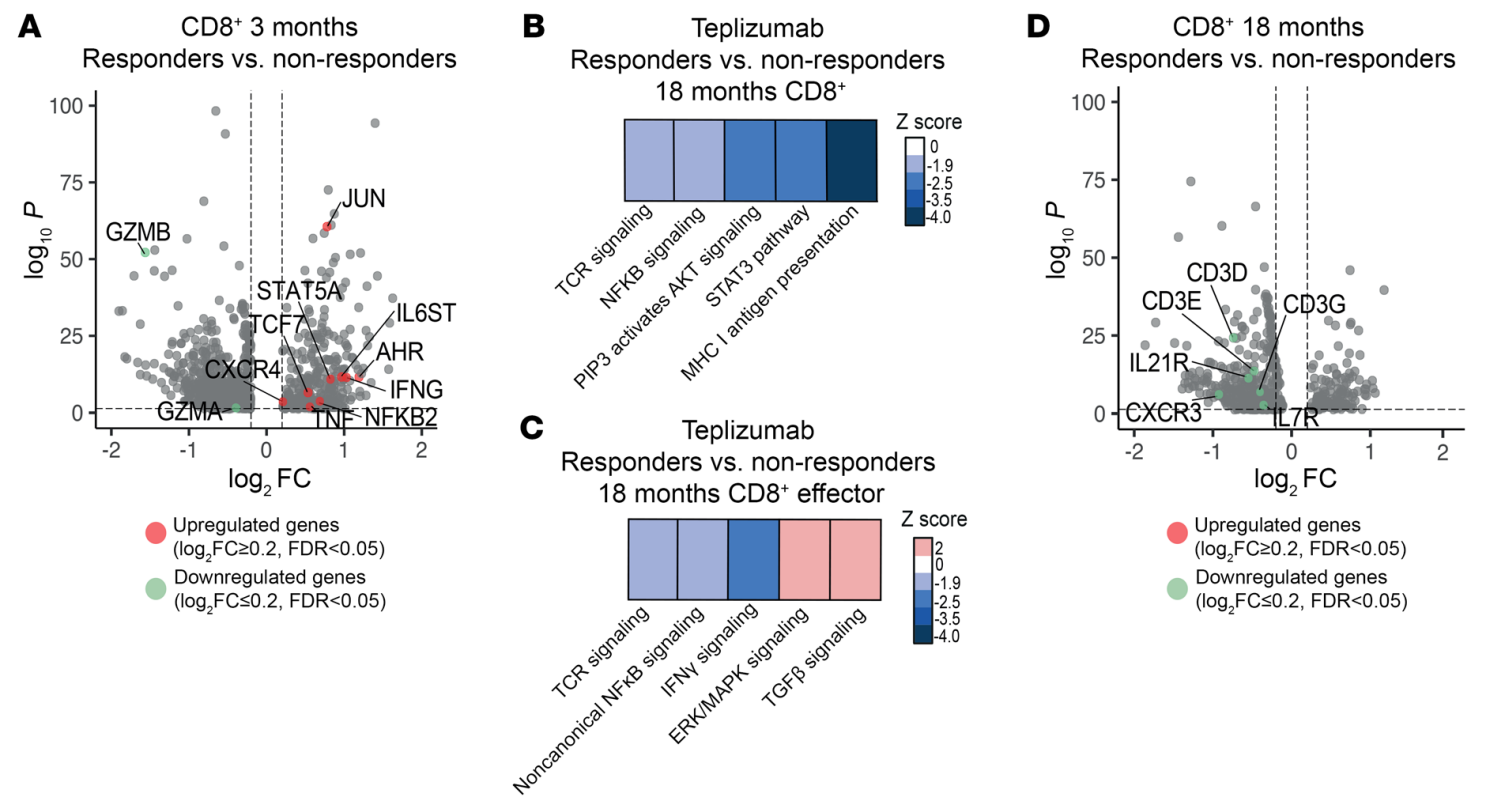
Changes across time in CD8^+^ T cells after teplizumab treatment in responders versus non-responders. (**A**) Volcano plot visualization of the DEGs in responders versus non-responders in CD8^+^ T cells at 3 months (*n* = 3 responders, *n* = 4 non-responders). The dashed lines identify average log_2_FC ≥ |0.2| and adjusted *P* value < 0.05. (**B** and **C**) Heatmap showing the *z* score of the pathways with statistically significant differences in the CD8^+^ effector and CD8^+^ T cell clusters at 18-month visit in the teplizumab group (*n* = 3 responders, *n* = 4 non-responders). The pathways were inferred based on the DEGs between responder and non-responder individuals and selected based on adjusted *P* < 0.05 by IPA software. Blue scale denotes grades of prediction of lower enrichment, while red scale indicates higher enrichment of the pathways based on the *z* score. (**D**) Volcano plot visualization of the DEGs in responders versus non-responders in CD8^+^ T cells at 18 months (*n* = 3 responders, *n* = 4 non-responders). The dashed lines identify average log_2_FC ≥ |0.2| and adjusted *P* value < 0.05.

We found 908 and 520 DEGs at 3 and 18 months, respectively, in a comparison of responders and non-responders in the teplizumab group (Supplemental Table 2). Interestingly, genes related to immune activation (*IFNG*, *TNF*, *IL6ST*) were upregulated in comparison with the non-responders at 3 months (Figure 4A). At 18 months, a pathway analysis comparing CD8^+^ T cells between responders and non-responders inferred reduced TCR signaling, NF-κB, STAT3, and AKT pathways, which are involved in cell activation, and reduced IFN-γ, TCR, and non-canonical NF-κB signaling but, in effectors, increased TGF-β pathways and ERK/MAPK signaling at 18 months (Figure 4, B and C). Consistent with the reduced enrichment in TCR signaling at 18 months, genes integral to TCR signaling, such as *CD3D*, *CD3G*, and *CD3E*, were reduced in responders versus non-responders at 18 months (Figure 4D). These findings again show a change in the characteristics of CD8^+^ T cells from 3 to 18 months after treatment resulting in reduced cell activation particularly among teplizumab responders.

**Figure 5:**
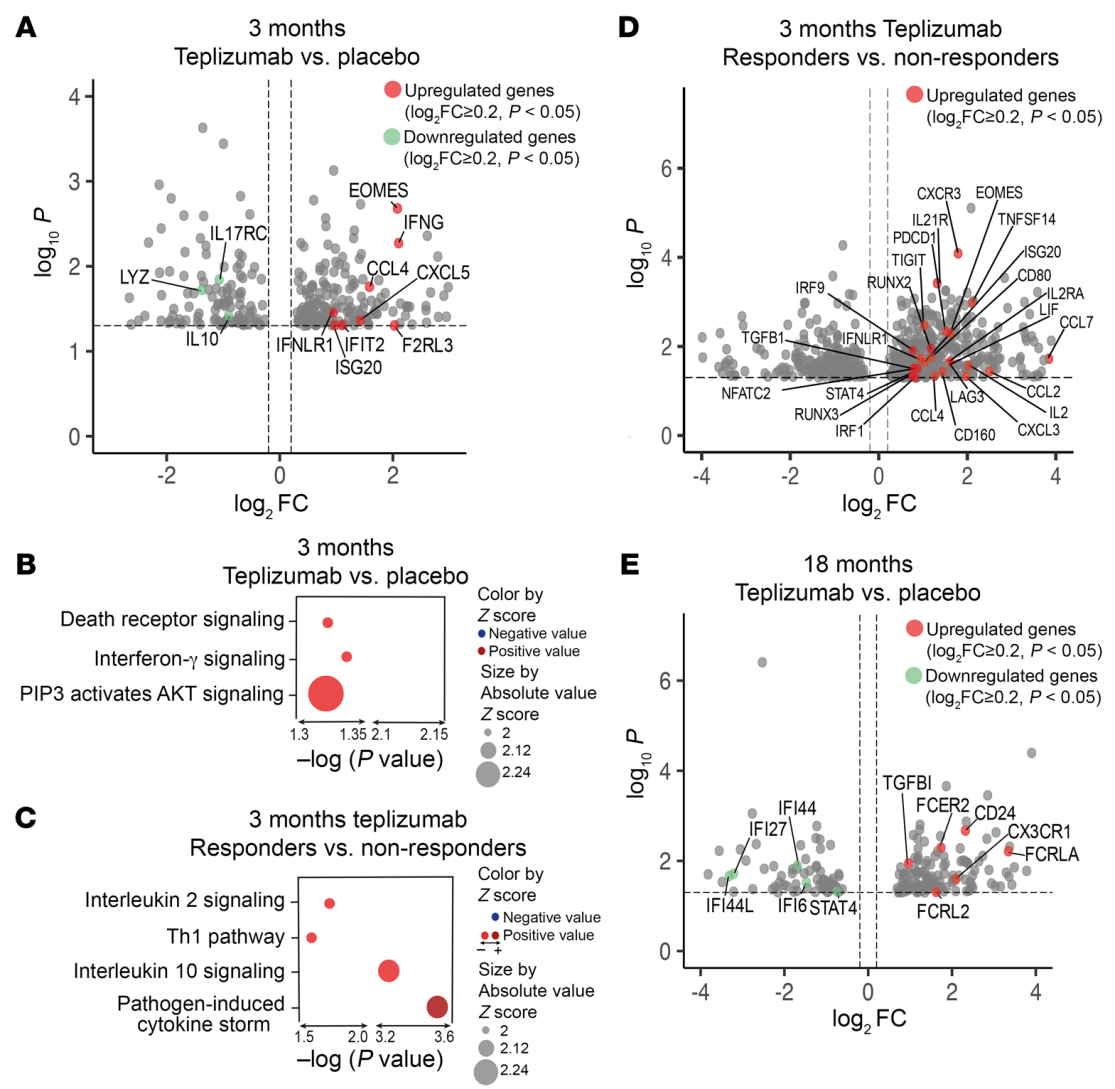
Cellular signatures and responses after teplizumab. (**A**) Volcano plot visualization of the DEGs in teplizumab versus placebo at 3 months (*n* = 10 teplizumab, *n* = 3 placebo) from bulk RNA-Seq. The dashed lines identify average log_2_FC ≥ |0.2| and *P* value < 0.05. (**B** and **C**) Bubble plots showing the *z* score and *P* values of the pathways with statistically significant differences in the teplizumab versus placebo groups at 3 months (*n* = 10 teplizumab, *n* = 3 placebo) (**B**) and in responders versus non-responders (*n* = 6 non-responders, *n* = 4 responders) in the teplizumab group at 3 months (**C**). (**D**) Volcano plot visualization of the DEGs in responders versus non-responders at 3 months from bulk RNA-Seq (*n* = 6 non-responders, *n* = 4 responders). The dashed lines identify average log_2_FC ≥ |0.2| and *P* value < 0.05. (**E**) Volcano plot visualization of the DEGs in teplizumab versus placebo at 3 months (*n* = 10 teplizumab, *n* = 3 placebo) and at 18 months (*n* = 2 teplizumab, *n* = 3 placebo) from bulk RNA-Seq. The dashed lines identify average log_2_FC ≥ |0.2| and *P* value < 0.05.

To validate these findings, we compared, by bulk RNA-Seq, gene expression and pathway analysis in total PBMCs from a second subcohort of participants using samples collected from additional patients at baseline (*n* = 9 teplizumab, *n* = 5 placebo), 3 months (*n* = 10 teplizumab, *n* = 3 placebo), and 18 months (*n* = 3 placebo, *n* = 2 teplizumab) (Supplemental Table 3). We again filtered out the differences between the participants before treatment. Among the 309 DEGs at 3 months (Figure 5A), we identified increased expression of *EOMES*, *IFNG*, *CXCL5*, and *CCL4*, related to immune activation. Our pathway analysis also showed enrichment of IFN-γ signaling and AKT signaling (Figure 5B). When teplizumab-treated responders (*n* = 4) and non-responders were compared at 3 months, we found increased expression of *EOMES*, as well as *PDCD1*, *LAG3*, and *TIGIT* (Figure 5D), and enrichment in IL-2, IL-10, and cytokine signaling (Figure 5C). At 18 months, *TGFB1* expression remained increased, but expression of IFN-γ pathway genes like *IFI44*, *IFI6*, and *IFI27* was reduced (Figure 5E). These findings confirm an enrichment in genes and pathways associated with immune activation at 3 months followed by expression of genes related to immunoregulation and exhaustion at 18 months.

We also compared DEGs in the patients before and after teplizumab with those in a published database from healthy controls whose scRNA-Seq had been analyzed at our center (*n* = 6, 4 males, 2 females, mean age = 11.3 years) (14) (Supplemental Figure 4). There were more than 2,000 DEGs and 120 pathways that were significantly different with IPA analysis between the patients and healthy controls (HCs) when compared before and 18 months after treatment in both study arms. In comparison with patients at enrollment, there were fewer differences after treatment with teplizumab than with placebo in both CD4^+^ and CD8^+^ T cells, suggesting that changes that had occurred in the placebo group over 18 months were fewer with teplizumab treatment (Supplemental Table 4).

### IL-7 receptor expression and long-term responses to teplizumab.

**Figure 6:**
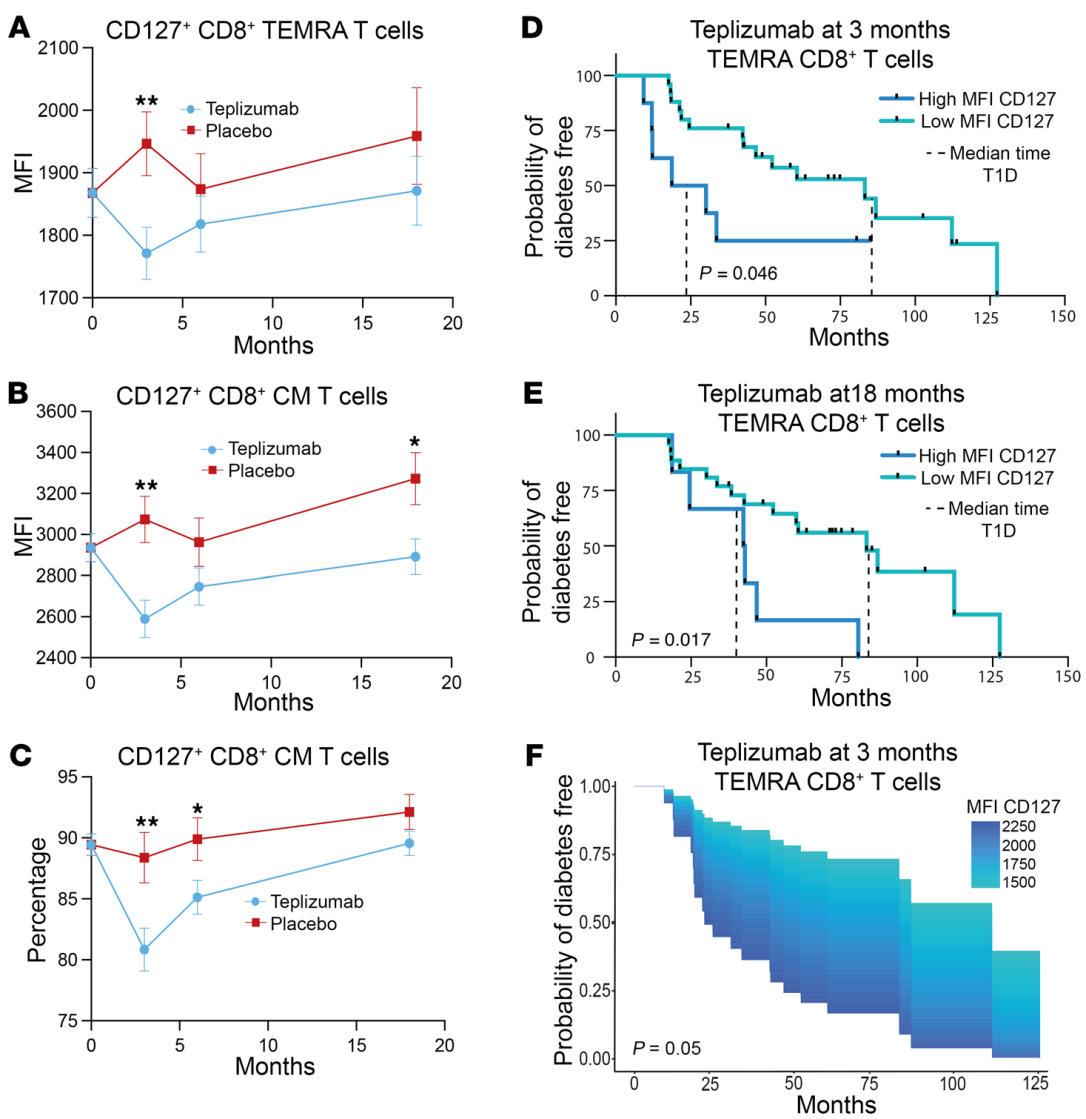
CD127 expression levels predict response to teplizumab. (**A**–**C**) Flow cytometry data of TN10 trial showing the difference in the MFI of CD127^+^ EMRA CD8^+^ T cells (TEMRA CD8^+^ T cells) (**A**), MFI CD127^+^ CM CD8^+^ T cells (**B**), and percentages of CD127^+^ CM CD8^+^ T cells (**C**) between teplizumab and placebo at different time points (*P < 0.05, **P < 0.01, *n* = 38 teplizumab, 28 placebo). The means + SEM from the mixed model are shown. (**D**) Kaplan Meier curves showing the difference between the median time to onset of T1D in teplizumab-treated patients with values above or below the median of the MFI of CD127 on TEMRA CD8^+^ cells in the placebo-treated patients at 3 months. The median times to stage 3 T1D were 83.2 months in the teplizumab-treated patients with MFI CD127 on CD8^+^ TEMRA cells below the median (*n* = 25) and 24.5 months for those above the median (*n* = 8) (log-rank *P* = 0.046). (**E**) The same comparison based on the median MFI of CD127 on CD8^+^ TEMRA cells in teplizumab-treated patients at 18 months. The median times to stage 3 T1D are 83.2 months with MFI below the median (*n* = 26) and 42.6 months with values below the median (*n* = 6) (log-rank *P* = 0.017). (**F**) Cox regression model showing the association between the time to diagnosis with stage 3 T1D based on a quantitative measure of the MFI of CD127 on TEMRA CD8^+^ cells in the teplizumab-treated group at 3 months (*n* = 32) (*P* = 0.05).

We found reduced expression of *IL7R* in CD4^+^ and CD8^+^ T cells from teplizumab- versus placebo-treated patients at 3 and 18 months (Figures 2 and 3 and Supplemental Table 1) and in the teplizumab-treated responder versus non-responder CD8*^+^* cells by scRNA-Seq at 18 months (Figure 4D and Supplemental Table 2). We confirmed these findings by flow cytometry and observed statistically significant reduced expression of CD127 in effector memory RA (TEMRA) and central memory (CM) CD8^+^ T cells at 3 months (*P* < 0.01) and a reduced frequency of CD127^+^ CM CD8^+^ T cells at 3 and 6 months (*P* < 0.01, *P* < 0.05) (Figure 6, A–C). To understand the clinical relevance of this finding, we compared the time to diagnosis with stage 3 T1D in teplizumab-treated patients with CD127 expression below (low MFI) and above the median expression of CD127 on EMRA CD8^+^ T cells from placebo-treated patients at 3 and 18 months (Figure 6, D–F). Teplizumab-treated patients with lower expression of CD127 on EMRA CD8^+^ T cells at 3 and 18 months had a longer median time to diagnosis with stage 3 disease (log rank *P* = 0.046 and 0.017), respectively (Figure 6, D and E). In a Cox regression analysis without censoring the data, lower expression of CD127 on EMRA CD8^+^ T cells at 3 months was associated with a slower progression of the disease (*P* = 0.05) (Figure 6F). Even when dividing the teplizumab-treated patients by the median expression of CD127 on EMRA CD8^+^ T cells in the teplizumab-treated group, at 3 and 18 months, those with lower expression had longer median times to progression to stage 3 disease (at 3 months, below the median: 86.9 months [*n* = 17], above median [*n* = 16] versus 31.9 months [*P* = 0.04, log-rank]; at 18 months, below the median: 87 months [*n* = 16], above the median 47 months [*n* = 16][*P* = 0.092 log-rank]).

### Differentiation of CD8^+^ T cells is affected by teplizumab treatment.

**Figure 7:**
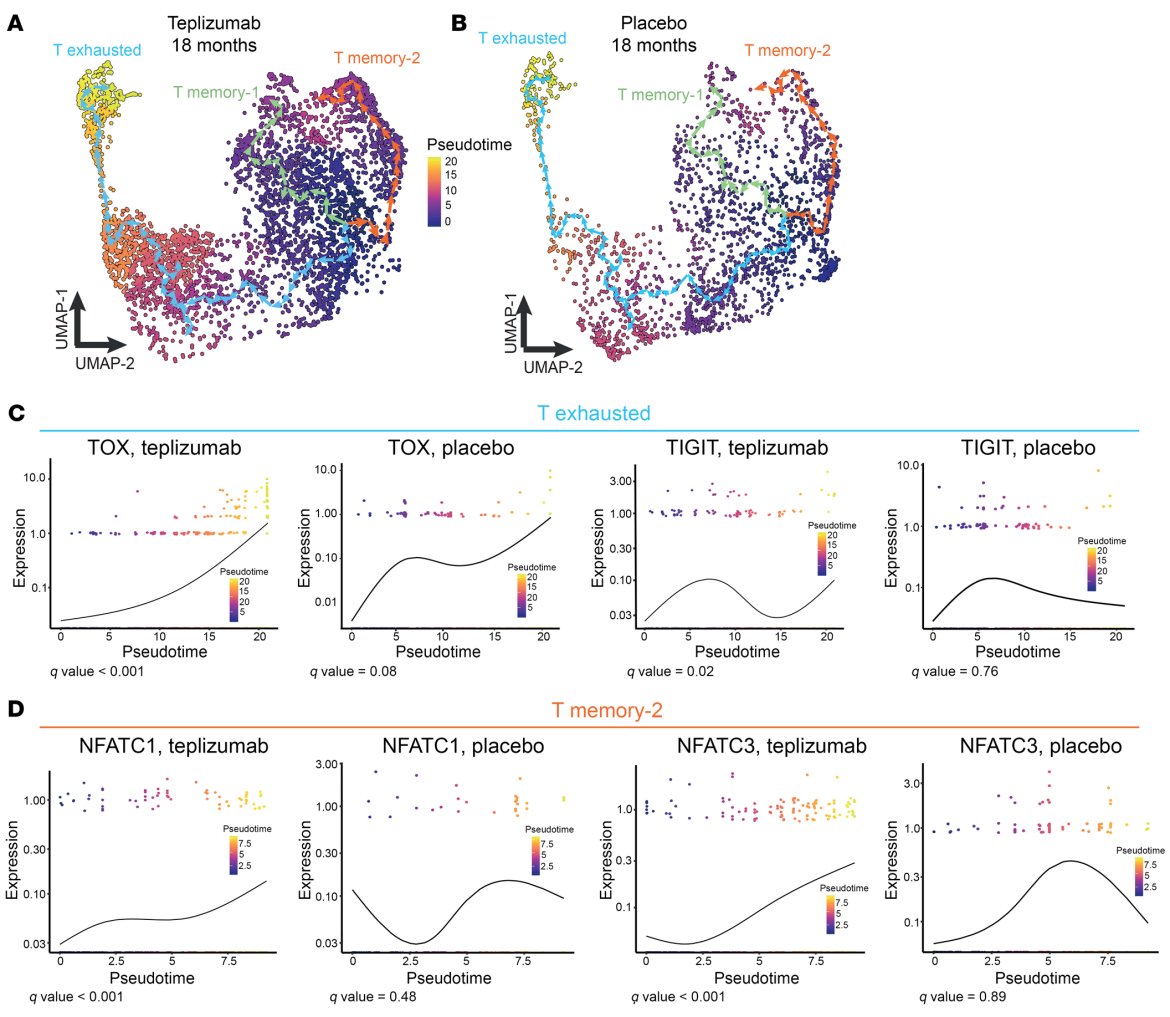
Pseudotime analysis of CD8^+^ T cells in the TN10 trial at 18 months. (**A** and **B**) 2D UMAPs showing the pseudotime analysis of CD8^+^ T cells in the teplizumab and placebo groups at 18 months (*n* = 7 teplizumab, *n* = 6 placebo). Cells are colored based on the pseudotime values. (**C** and **D**) Gene expression dynamics across the pseudotime in the teplizumab and placebo groups in the exhausted and memory-2 branches of differentiation (*n* = 7 teplizumab, *n* = 6 placebo).

These studies indicated that there were changes induced by teplizumab treatment that evolved after treatment. CD8^+^ T cells are thought to be the effectors in the disease, and therefore, to identify how drug treatment changed the trajectory of CD8^+^ T cell differentiation, we performed a pseudotime analysis of these cells at 18 months in the 2 treatment arms. Cells were pseudo-colored in each uniform manifold approximation and projection (UMAP) embedding based on their pseudotime values from 0 to 20 in the effector branch and from 0 to 7 in the memory-1 and memory-2 branches, and expression in associated plots showed relative cell distribution frequency of the genes as a function of pseudotime (Figure 7, A and B, and Supplemental Table 5). A pseudo-temporal ordering of cells from Monocle on the co-embedded CD8^+^ T cells at 18 months in both treatment groups was extracted, and a developmental trajectory was estimated to the latest stages of differentiation. We assigned functional features based on known gene expression (15).

Among the teplizumab arm, we found a preponderance of exhausted T cells with a statistically significant change in *TOX* and *TIGIT* (*q* value = 0.001 and 0.02, respectively), but this was not found in the placebo arm (Figure 7C). This also corresponded with the overall shift in the T memory-2 branch, where NFATC1 and NFATC3 (*q* value < 0.001) were also found to have statistically significant differences in comparison with the placebo (Figure 7D). In the memory-1 branch, we found statistically significant differences in CXCR4 and FOXO3 in the teplizumab group (Supplemental Figure 5). These results were consistent with our DEG and pathway analyses suggesting features of exhaustion and regulation among CD8^+^ T cells 18 months after treatment.

### Effects of teplizumab treatment on the frequency and repertoire of antigen-specific CD8^+^ T cells.

**Figure 8:**
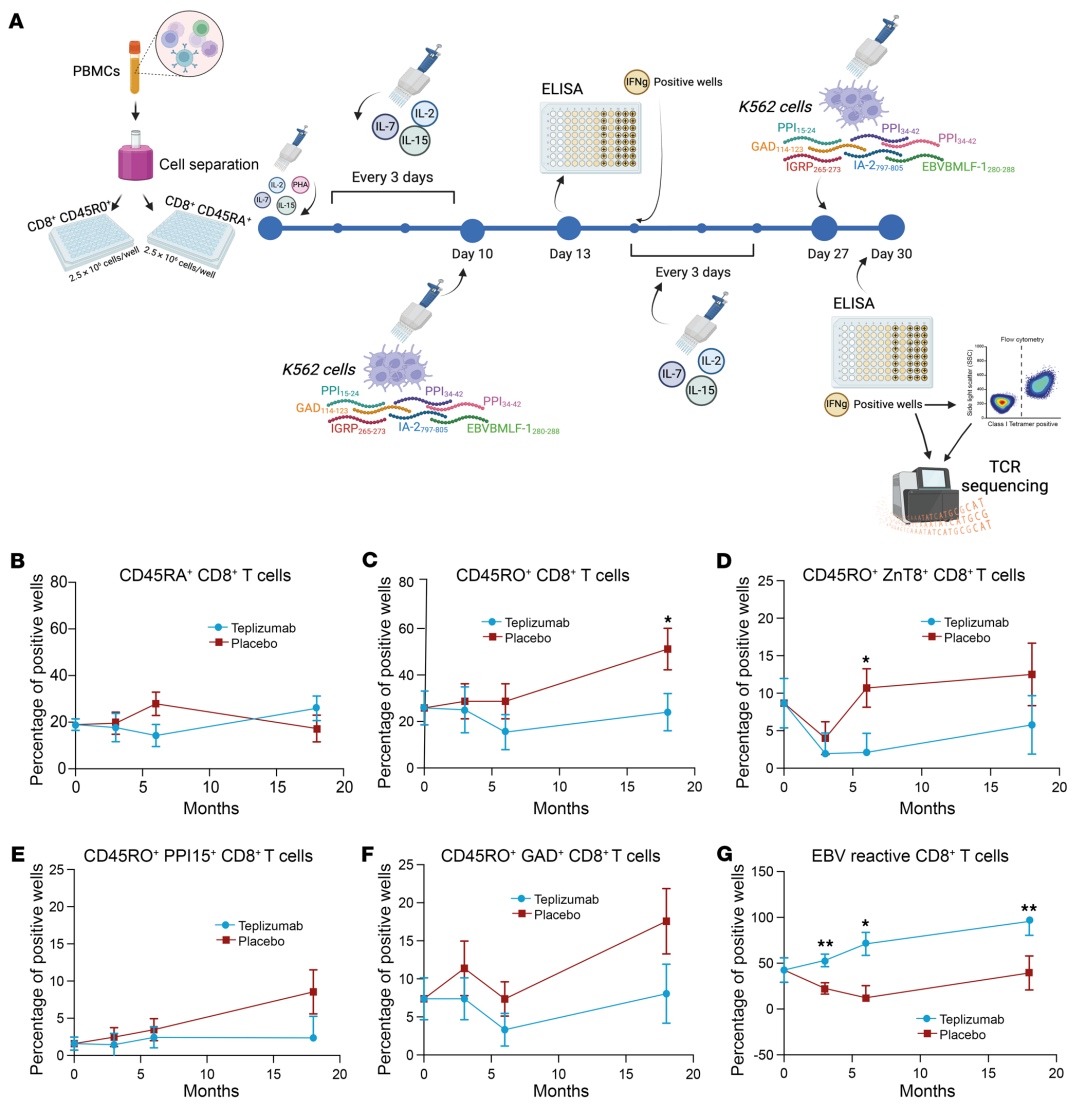
Effects of teplizumab treatment on antigen-reactive CD8^+^ T cells. (**A**) Schematic experiment design for the T cell libraries. (**B** and **C**) CD8^+^ T cell library analysis of antigen-specific T cells. Total antigen-reactive wells from libraries of CD45RA^+^ (**B**) or CD45RO^+^ (**C**) T cells from teplizumab-treated (*n* = 10) and placebo-treated (*n* = 12) patients (*P = 0.025 by repeated-measures ANOVA with correction for the baseline). (**D**–**G**) Analysis of frequency of library wells reactive to each of the peptides used in the CD8^+^ T cell libraries: CD45RO^+^ ZnT8-reactive CD8^+^ T cells (**D**), CD45RO^+^ PPI15-reactive CD8^+^ T cells (**E**), CD45RO^+^ GAD65-reactive CD8^+^ T cells (**F**), and CD45RO^+^ EBV-reactive CD8^+^ T cells (**G**) from teplizumab-treated (*n* = 10) and placebo-treated (*n* = 12) individuals (*P < 0.05, **P < 0.01 by repeated-measures ANOVA with correction for the baseline). Mean ± SEM from the mixed model is shown.

We analyzed the effects of drug treatment on autoantigen-specific CD8^+^ T cells. We prepared CD8^+^ T cell libraries and compared the frequency of antigen-reactive cells (detected by IFN-γ production) in the 2 treatment arms using published methods to determine their relative frequency among antigen-experienced (i.e., CD45RO^+^) and -naive (i.e., CD45RA^+^) CD8^+^ T cells (16) (Figure 8A). There was an increase in the frequency of wells reactive with autoantigens among the CD45RO^+^CD8^+^ T cells at 18 months that was greater than in the teplizumab treatment arm (from 25.8% ± 7.3% of wells at baseline to 51.1% ± 9% in placebo vs. 24.0% ± 8% in teplizumab; difference in least-square means 27.2% ± 11.3%, *P* = 0.025). The frequency of autoantigen-reactive CD45RA^+^ cells did not increase in the placebo treatment arm over time and was not different in comparison with the teplizumab-treated patients (Figure 8, B and C).

To identify which autoantigen reactivities accounted for these changes, we compared the frequencies of the CD8^+^CD45RO^+^ T cells that were reactive to each of the 6 peptides that were used for challenge. The frequency of wells with reactivity to ZnT8 showed a statistically significant increase at month 6 in placebo- versus teplizumab-treated patients (difference in least-square means for teplizumab vs. placebo, 8.6% ± 3.4%, *P* = 0.019), and likewise there was an increase in the frequency of wells responsive to PPI15 (difference in least-square means for teplizumab vs. placebo, 6.2% ± 4.1%, *P* = 0.15) and GAD (difference in least-square means for teplizumab vs. placebo, 9.5% ± 5.3%, *P* = 0.085) at month 18 (Figure 8, D–F). The frequency of the RA^+^ cells that were reactive to the same peptides did not change significantly.

Curiously, unlike the frequency of the wells with autoantigen-reactive CD8^+^ T cells, which was lower in the teplizumab-treated patients, the frequency of the wells with EBV-reactive (i.e., EBNA) CD8^+^ T cells increased (from 42.6% ± 13.4% of wells at baseline to 95.6% ± 15.2% of wells at month 18) and was statistically significantly higher than in placebo at months 3 (*P* = 0.0025), 6 (*P* = 0.01), and 18 (*P* = 0.004) in the 5 teplizumab-treated patients who were known to be EBV seropositive at study entry compared with the 4 EBV seropositive placebo-treated patients (Figure 8G). (We pooled both CD45RO^+^ and RA^+^ cells because some EBV memory cells may express CD45RA [i.e., TEMRAs].)

These effects on autoantigen-reactive cells may have entailed depletion, or prevention of expansion, of existing cells, or the appearance of new cells. To determine whether antigen-reactive CD8^+^ T cells that were present before treatment were eliminated, we sorted antigen-specific T cells from the T cell libraries with class I MHC tetramers that were loaded with the diabetes antigen peptides from positive wells from different study visits and tracked the sequences of the Vβ chains from those cells before and after treatment. We did not find sharing of Vβ chain sequences between patients when we compared the sequences of tetramer-sorted cells reactive to the same peptides, i.e., the sequences were private. We determined the relative frequency of the sequences at each study visit in the wells that showed positive responses to the same peptide. In both treatment groups, some sequences were present at each of the study visits, whereas new sequences were detected at later times. In addition, some sequences that were present at the baseline were not detected at later visits. In the drug treatment group, some sequences persisted at high frequency whereas others declined (Supplemental Figure 6, A–C). We found the same general pattern of TCR Vβ chain sequences in the placebo group — there was emergence of novel sequences along with the persistence of certain more dominant sequences (Supplemental Figure 6, D–F). Based on our findings of reduced IL-7R (CD127) on CD8^+^ T cells in the teplizumab-treated patients, these data suggest that, rather than depletion, teplizumab treatment blocked the growth of the autoantigen-reactive cells that were present before treatment but expanded in the placebo group.

## Discussion

We report that a single 14-day course of teplizumab treatment delays progression from stage 2 to clinical (stage 3) T1D, with 36% of patients remaining disease free or having onset after more than 5 years. The outcome in these patients suggests induction of operational tolerance in some patients, since disease prevention did not require continuous drug treatment or prolonged immune suppression. Previous studies described a partial exhaustion transcriptional and cellular phenotype in CD8^+^ T cells within 6 months of drug administration, most likely resulting from the partial agonist signal of the anti-CD3 mAb (2). However, these phenotypes resolved by 18 months. We found changes in subsets of circulating CD4^+^ and CD8^+^ T cells in patients at 3 months and even 18 months after they were treated with teplizumab. We used scRNA-Seq to identify the effects of drug treatment on subsets of T cells and were able to distinguish the effects in drug-treated clinical responders, who remained without stage 3 T1D for at least 5 years. At 3 months, there was enrichment of pathways involving IFN-γ and other cytokine signaling in CD4^+^ and CD8^+^ T cells. However, there were cell subset–specific differences: CD4^+^ effector T cells had enhanced TCR signaling, whereas in CD8^+^ effector T cells this pathway was reduced. At 18 months, JAK signaling persisted in CD4^+^ T cells, while IFN-γ signaling was reduced in CD8^+^ T cells and TGF-β signaling was increased. When we compared clinical responders versus non-responders, both CD4^+^ and CD8^+^ cells showed diminished TCR and other signaling pathways at 18 months. We confirmed these patterns with bulk RNA-Seq on PBMCs from a second cohort, revealing enrichment in IFN-γ and AKT signaling at 3 months, with upregulation of genes related to exhaustion (*EOMES*) and immunoregulation (*TGFB1*) at 3 months and 18 months, respectively. With a pseudotime analysis we identified changes in the trajectory of CD8^+^ T cell development and enhanced expression of genes associated with exhaustion in effector and memory CD8^+^ T cells with teplizumab. These induced changes in CD8^+^ T cells appear to identify patients with the most robust responses to the drug even when other features (e.g., TIGIT/KLRG1 expression) have resolved. *IL7R* was reduced in expression in CD8^+^ T cells from teplizumab-treated responders, and the level of expression of CD127 on CD8^+^ T cells was a marker of clinical responses. Consistent with this, we found reduced expansion of antigen-specific CD8^+^ memory T cells with teplizumab treatment.

The extended effects of teplizumab developed without persistence of the drug. Teplizumab binds to and causes reduced expression of the CD3 molecule, peaking on the last dosing day and declining rapidly from T cell surfaces by 28 days after the treatment (17). Therefore, the alterations at 3 and 18 months reflect evolving cellular characteristics in the T cells after exposure. In murine studies, bivalent non-FcR-binding anti-CD3 mAb delivered a signal in T cells that did not induce the highly phosphorylated form of CD3ζ, and led to T cell anergy (18). This signal may mimic the weak agonist signals seen by autoreactive T cells when they encounter self-peptides in healthy individuals. Our previous studies had shown a preferential effect of teplizumab on CD8^+^ T cells, but in these new studies, even 18 months after treatment, in the CD8^+^ T cells, there was increased development of cells with transcriptional features of exhaustion and immunoregulation such as TOX and TGF-β signaling, but reduced TCR signaling (19–24). Interestingly, we identified decreased TXNIP expression in total CD8^+^ cells at the 18-month visit, raising the possibility that, in addition to its proposed effects on β cells, verapamil may also have effects on CD8^+^ T cells (25). The reasons for the differential effects on CD4^+^ and CD8^+^ T cells or among T cell subsets are not clear at this time. Further studies of the molecular mechanisms and the genetic differences between responders and non-responders may shed light on mechanisms that can explain the outcome of cells after treatment.

A feature of CD8^+^ T cells from drug-treated patients and the responders was reduced expression of the *IL7R* gene and CD127 by flow cytometry. There was reduced expression on TEMRA CD8^+^ cells at 3 months and on CM CD8^+^ T cells at 3 and 18 months with reduced frequency of the CD127^+^ CM CD8^+^ T cells at 3 and 6 months. *IL7R* is needed for the expansion and maintenance of memory CD8^+^ T cells and supports antigen-specific expansion and effector function (26). It can also facilitate antigen-independent T cell proliferation in settings such as lymphopenia with a greater effect on CD8^+^ than CD4^+^ T cells (27). Long-lived memory T cells require IL-7 signals for maintenance (28, 29). Thus, our finding that there was reduced expansion of autoantigen-specific CD8^+^ T cells with teplizumab treatment was consistent with these known actions of the cytokine. Our flow and RNA-Seq studies show effects in different CD8^+^ T cell subsets, including subsets that contain autoantigen-reactive cells. This persistent feature of teplizumab treatment may also account for the clinical responses, since we found that patients with lower expression of CD127 on CD8^+^ TEMRAs at 3 or 18 months had extended responses to teplizumab.

Curiously, not all CD8^+^ T cells were equally affected. EBV-reactive CD8^+^ T cells increased in frequency even through month 18. This was not the result of persistent EBV viremia, since in those participants in whom EBV DNA became detectable after teplizumab treatment, the EBV DNA levels decreased to undetectable levels between day 43 and day 134 (mean day 77) after the start of study drug (all of the patients in whom EBV-reactive T cells were measured were EBV seropositive at entry, but EBV viral loads were detectable during treatment in 2 of the 5 patients who were studied). The reason for the continued increase in frequency is uncertain. We cannot exclude that there was a low, clinically undetectable level of viral reactivity. Additional studies of T cells that are reactive with other latent viruses may help to address the reasons for the response to EBV. The selective effects on autoantigen-reactive cells may reflect other features of the cells, such as their TCR avidity or IL-7–independent growth. Thus there appear to be selective effects of teplizumab that may explain the therapeutic efficacy in the absence of signals of infection complications in this or other studies with the drug (5, 6).

The mAb did not simply deplete autoreactive T cells at 3 or even 18 months. We cannot be certain about the absolute frequency of autoantigen-reactive T cells, and therefore cannot exclude partial depletion, but our data show clearly that sequences that are found before treatment reappear at later times. Our analysis of DEGs and IPAs in comparison with healthy controls and with patients at the baseline and over time suggests that teplizumab may have reduced changes in CD8^+^ T cells during disease progression but did not induce a more “normal” transcriptional pattern.

We found reduced *IL7R*/CD127 on the CD8^+^ cells, suggesting an induced mechanism that prevents the expansion of autoantigen-reactive T cells because they could not utilize IL-7, a cytokine needed for memory T cell growth. It is also possible that the reappearance of the TCR sequences arises from a common stem cell that is resistant to effects of teplizumab (30, 31). As autoantigen-reactive memory CD8^+^ T cells are thought to be the important effectors of β cell killing, these findings suggest how direct activity of the mAb and its persistent effects may explain the lasting effects on disease prevention. Further studies of the antigen-specific cells after teplizumab treatment might identify the basis for their growth failure.

There are limitations to this analysis. We cannot be certain that the changes we observed in the polyclonal T cells such as the expression of markers of exhaustion or even their developmental trajectory were induced in the diabetes antigen–specific cells. In addition, we do not know the absolute number of auto- or viral antigen–reactive CD8^+^ T cells in the peripheral blood. Our library studies involved 2 rounds of polyclonal stimulation and required growth in vitro to be identified. The reduced expression of CD127 may have limited the expansion of the autoreactive cells in the libraries when the initial expansion was performed since IL-7 was added to the wells. The features that may have existed in vivo may have been altered in vitro, and therefore reduced frequency of antigen-specific cells may reflect failure of growth rather than depletion. We focused on studies of T cells because they are the targets of teplizumab. There may have been changes in other cell types that affect the function of relevant T cells. Our definition of responders is relative and could be affected by personal or other immunologic features that we did not investigate. Finally, the number of participants we were able to study was limited but included a validation cohort and complementary methods to confirm our findings. Further analyses with larger numbers of patients followed for more extended periods of time would be helpful in understanding the mechanisms for maintaining operational tolerance.

In summary, our study has identified statistically significant alterations in CD4^+^ and CD8^+^ T cells 3 months after teplizumab treatment and when the drug is no longer detectable. There was T cell activation at 3 months, followed by evolving changes, which include the induction of genes associated with T cell exhaustion, immune regulation, and impaired T cell expansion, even 18 months after a single course of drug, and at a time when the previously identified phenotypes of cell exhaustion are gone. The treatment effectively curbs the expansion of autoantigen-reactive CD8^+^ T cells, although it does not globally suppress immune responses, allowing viral antigen–reactive T cells to continue to expand. These early responses appear to distinguish those with persistent drug effects. Our findings suggest that the partial agonistic signal triggered by teplizumab sets in motion changes in T cells that unfold over time and serve as a check against disease progression. Further studies comparing patients protected from diabetes for more extended periods of time with healthy controls may identify pathways that are essential for the maintenance of tolerance. These studies describe how teplizumab might be strategically employed to induce responses in autoimmune diseases like T1D and features of operational tolerance.

## Methods

### Sex as a biological variable.

Among the 76 patients enrolled and followed (Supplemental Figure 1), 42 were male (25 in the teplizumab and 17 in the placebo group) and 34 were female (19 in the teplizumab group and 15 in the placebo group). Sex did not have a statistically significant effect on treatment outcome. Antigen-reactive CD8^+^ T cells were analyzed in samples from 7 male and 3 female teplizumab-treated and 6 male and 6 female placebo-treated study participants. The patients were all White.

### Patients and samples.

Clinical data from the TN10 teplizumab prevention trial (Supplemental Figure 1) were followed for progression to diagnosis of T1D, which was made using American Diabetes Association criteria (32). (The data cutoff was July 2023.) Teplizumab-treated patients (*n* = 17/44, 39%) who were diagnosed after 60 months after treatment or were not diagnosed after 60 months after treatment with stage 3 T1D were designated as responders. Six (of 32) placebo-treated patients had stage 3 diagnosis after 60 months (19%).

Flow cytometry analyses were done on 66 individuals of the original 76 participants at the core laboratory of the Immune Tolerance Network (Benaroya Research Institute). For CD8^+^ T cell libraries and scRNA-Seq, samples from 22 participants (10 teplizumab treated, 12 placebo treated) from HLA-A2.1^+^ individuals were used. From these, 14 (7 teplizumab and 7 placebo) were used for scRNA-Seq. Samples were also obtained from an additional 15 participants (10 teplizumab, 5 placebo) for bulk RNA-Seq. The data reported with each analysis represent the total number of usable samples. HLA genotyping was done by the TrialNet Core laboratory (Benaroya Research Institute). We analyzed samples from the patients from enrollment and at 3, 6, and 18 months. Beyond that time (i.e., by month 27), half of the placebo-treated patients had been diagnosed with stage 3 T1D. The following reagents were used for flow analysis: from Becton Dickinson, anti-CD45RA (catalog 564442), anti-CD45RO (catalog 564290), and anti-CD4 (catalog 564419); from BioLegend, anti-CD127 (catalog 351328), anti-CD3 (catalog 317322), anti–PD-1 (catalog 329950), and anti-CCR7 (catalog 353232); and from eBioscience, anti-EOMES (catalog 12-4877-42).

### Single-cell RNA-Seq processing and analysis.

PBMCs (25 × 10^5^ cells per patient per visit) were reconstituted with wash buffer and incubated with Human TrueStain Fc Blocker (BioLegend) for 10 minutes. TotalSeq antibodies were applied without washing of the cells and were incubated at 4°C for 30 minutes. After 3 rounds of washing, samples were pooled into one tube based on cell counts, and super-loaded onto the 10× Genomics Chromium Chip. The loaded Chip was generated and processed by the Yale Center for Genome Analysis. The cDNA samples were used to construct 2 types of cDNA libraries, according to the steps outlined in the user guide: gene expression libraries and cell surface hashtag libraries. cDNA libraries were then sequenced on an Illumina NovaSeq 6000 platform.

### Data processing of raw sequencing reads and scRNA-Seq analysis.

Raw sequencing reads were demultiplexed using Cell Ranger mkfastq pipeline to create FASTQ files. Cell Ranger count pipeline (v3.1) was used in order to perform alignment (using STAR), filtering, barcode counting, and unique molecular identifier (UMI) counting. The samples from 7 teplizumab- and 7 placebo-treated patients were analyzed by scRNA-Seq. Approximately 20,000 PBMCs were submitted for sequencing to the Yale Center for Genome Analysis. The gene-cell barcode matrices were used for further analysis with the R package Seurat (Seurat development version 4.0.2 and 5.0.3) with additional use of the packages dittoSeq, harmony, scCustomize, SCpubr, Monocle 3, pheatmap, and EnhancedVolcano. Demultiplexing was done using HTODemux with automatic thresholding. Cells were filtered if they were classified as doublets or negative for hashtag antibody based on the demultiplexing results, or if they had fewer than 200 features, or if greater than 5% mitochondrial RNA was detected. After removal of likely multiples and low-quality cells, the gene expression levels for each cell were normalized with the NormalizeData function in Seurat followed by the integration of the single-cell data. The integrated data were scaled, and the principal analysis was performed. Clusters were identified using the FindNeighbors and FindClusters Seurat functions. Batch correction was applied using RunHarmony function. Cell cluster identities were manually defined with the cluster-specific marker genes or known marker genes. The cell clusters were visualized using uniform manifold approximation and projection (UMAP) plots and completed using plot3D (https://plotly.com/python/3d-charts/). CD8^+^ and CD4^+^ T cells were reclustered separately at a resolution between 0.1 and 0.2 to obtain biologically meaningful clusters each respectively. Because of gene expression similarities, the naive CD4^+^ T cells could not be distinguished in the subset analysis. Subset functions were used to compare the different types of cells included in the study. Subset functions were used to compare the different types of cells included in the study. A MAST package was used to run the differential expression (DE) testing implemented in the FindAllMarkers function. Genes included in the comparison between teplizumab and placebo, or between responders and non-responders at baseline, were excluded if their average log_2_ fold change reached |0.2| or if their adjusted *P* value was less than 0.05 at subsequent time points (3 months and 18 months). IPA software and gene set enrichment analysis (GSEA) were used to analyze pathway expression. Ligand receptor analysis was done using the SCpubr package. To investigate the kinetics of gene expression during T and B cell differentiation, we performed single-cell trajectory analysis using the Monocle 3 package. The scRNA-Seq profiles of CD8^+^ T cells were used to reconstruct the single-cell trajectories for the different states. The group-specific marker genes were selected using the graph_test function. We used find_gene_modules to group genes into modules, which grouped genes with similar patterns of expression into modules. We then compared the differences between gene expression to the mean gene expression in the module (33). The cells were ordered pseudo-temporally using the reduceDimension and orderCells functions. The association between cells was tested by Moran’s I test available in graph_test function and plotted using the plot_genes_in_pseudotime function.

### Bulk RNA-Seq processing and analysis.

For RNA isolation, 100,000 to 500,000 PBMCs were preserved in Trizol, and RNA was extracted using the miRNAeasy micro kit (Qiagen). RNA-Seq libraries were produced using Illumina Truseq library preparation kits. The sequencing results were then demultiplexed and transformed into FASTQ format via the Illumina bcl2fastq pipeline. The sequencing reads were subsequently aligned to the Hg19 human genome using the splice-aware STAR aligner. Samples were analyzed using the DESeq2 package, and pathways were inferred using IPA software.

### T cell libraries.

We used previously described methods to identify the relative frequencies of autoantigen-reactive CD8^+^ T cells in T cell libraries (16). CD8^+^CD45RA^+^ and CD8^+^CD45RO^+^ cells were sorted from PBMCs and cultured in 96-well round-bottom plates (Corning) at 2.5 × 10^3^ cells per well in complete DMEM (supplemented with 10 mM HEPES, pH 7.3 [Corning], 0.1 mM nonessential amino acids [Gibco], 1 mM sodium pyruvate [Gibco], 50 U/mL penicillin [Gibco], 50 U/mL streptomycin [Gibco], and 5% human serum [Gemini Bio-products]) in the presence of 5 μg/mL phytohemagglutinin (MilliporeSigma catalog L1668), 20 U/mL IL-2, 20 ng/mL IL-7, and 20 ng/mL IL-15 with irradiated (45 Gy) allogeneic feeder cells (2 × 10^4^ cells per well) (recombinant human [rh] IL-7, catalog 581904; rhIL-15, catalog 570304; rhIL-2 PeproTech 200-02; BioLegend). Fresh cytokines were added every 3 days. On day 10, the libraries were screened by culturing of the total cells from each well (about 5 × 10^5^ cells), after washing, with K562 HLA-A2^+^4-1BBL^+^ cells (~10^5^ cells) (a gift from J. Riley, University of Pennsylvania, Philadelphia, Pennsylvania, USA), which were either unpulsed (DMSO) or pulsed for 2 hours with islet antigen peptide pools (PPI_15–24_, PPI_34–42_, GAD_114–123_, IGRP_265–273_, IA-2_797–805_, and ZnT8_186–194_) or viral peptides (EBVBMLF-1_280–288_) (Genscript). Culture supernatant was harvested on the third day, and IFN-γ was measured by ELISA. Wells that had IFN-γ levels greater than the mean + 3 SD of the control challenge (i.e., K562 cells without peptides) were further expanded by addition of IL-2, IL-7, and IL-15 every 2–4 days for another 14 days and then restimulated with irradiated K562 HLA-A2^+^4-1BBL^+^ cells pulsed with individual islet antigen peptides. IFN-γ was measured in supernatants after 3 days of stimulation. For IFN-γ, ELISA plates were coated with purified mouse anti-human IFN-γ (BD, catalog 551221). For detection of bound human IFN-γ, we used biotinylated mouse anti-human IFN-γ (Becton Dickinson, catalog 554550). Bound antibody was visualized with Pierce High-Sensitivity Streptavidin-Horseradish Peroxidase (catalog 21130), and developed with Ultra tetramethylbendidine (TMB) (Thermo Scientific, catalog 34029).

The wells that showed IFN-γ responses greater than mean + 3 SD of the control wells were designated as positive. The frequency of the antigen-specific wells represents the number of positive wells for each peptide or the sum of the wells for all the peptides/total number of CD45RO or CD45RA wells that were plated at the start of the assay.

### TCR sequencing and analysis.

Some wells that showed positive responses to peptides were stained with class I MHC tetramers conjugated to fluorophores (NIH Core, Emory University, Atlanta, Georgia, USA) and sorted on a Becton Dickinson Aria cell sorter. DNA was isolated from the sorted cells, in addition to selected wells that showed responses to peptides, and TCR Vβ chain sequencing was done at Adaptive Biotech. The frequency of TCR sequences in the tetramer+ cells and antigen wells with antigen reactive cells were determined using the scRepertoire and LymphoSeq toolkit in R (34, 35). Unique amino acid sequences of the Vβ chain of the individual tetramers were matched (after pooling of the RO and RA wells) with the sequences of each peptide-reactive well at different time points. We calculated the frequency of each Vβ chain sequence among the total sequences that were identified from the tetramer-sorted cells.

### Statistics.

Repeated measures were analyzed with correction for the baseline with a Fitzmaurice model (36) with SAS (v9.4, SAS Institute). Unless otherwise indicated, the data are reported as mean ± SEM. Kaplan-Meier curves were derived using the R function survfit from the “survival” package, or with PROC LIFETEST in SAS with P values computed using the log-rank test. A survival curve was also prepared using a Cox proportional hazards model implemented in the survival package, specifically utilizing the coxph function. To visualize and present these survival curves, we used the contsurplot package, making use of its plot_survival_area function. The analyses of DEGs, but not the bulk RNA-Seq or repeated-measures ANOVAs, were corrected for multiple comparisons. *P* values less than 0.05 were considered statistically significant.

### Study approval.

The details of the clinical trial have been published previously (1, 37). The trial was conducted from July 2011 through November 2018 at sites in the United States, Canada, Australia, and Germany. Institutional review board approval was obtained at each participating site. The participants, their parents, or both provided written informed consent or assent before trial entry. Eligible participants were found to have stage 2 T1D (i.e., ≥2 autoantibodies and dysglycemia). The trial was registered: ClinicalTrials.gov NCT01030861.

### Data availability.

The supporting data for the displayed analyses are available in the Supporting Data values file. The raw transcriptomic data were deposited in the NCBI’s Gene Expression Omnibus database (GEO GSE271063). Flow cytometry data are available in the National Institute of Diabetes and Digestive and Kidney Diseases repository (https://repository.niddk.nih.gov).

## Author contributions

ALD and PPH performed experiments, analyzed data, and wrote the manuscript. Order of first authors is based on contributions to these tasks. SC, PC, PSL, SAL, CL, and GK processed samples and acquired data. AJM and JST revised and edited the manuscript. KCH led the clinical trial, designed the studies, acquired samples, analyzed data, and wrote the manuscript.

## Supplementary Material

Supplemental data

ICMJE disclosure forms

Supplemental table 1

Supplemental table 2

Supplemental table 3

Supplemental table 4

Supplemental table 5

Supporting data values

## Version 1 08/13/2024

In-Press Preview

## Version 2 09/17/2024

Electronic publication
