# Supplementary material for: Teplizumab induces persistent changes in the antigen-specific repertoire in individuals at risk for type 1 diabetes
Source: J Clin Invest. 2024 Aug 13;134(18):e177492. doi: 10.1172/JCI177492 (PMC11405034; doi:10.1172/JCI177492)
Supplement: Supplemental data [file jci-134-177492-s264.pdf]

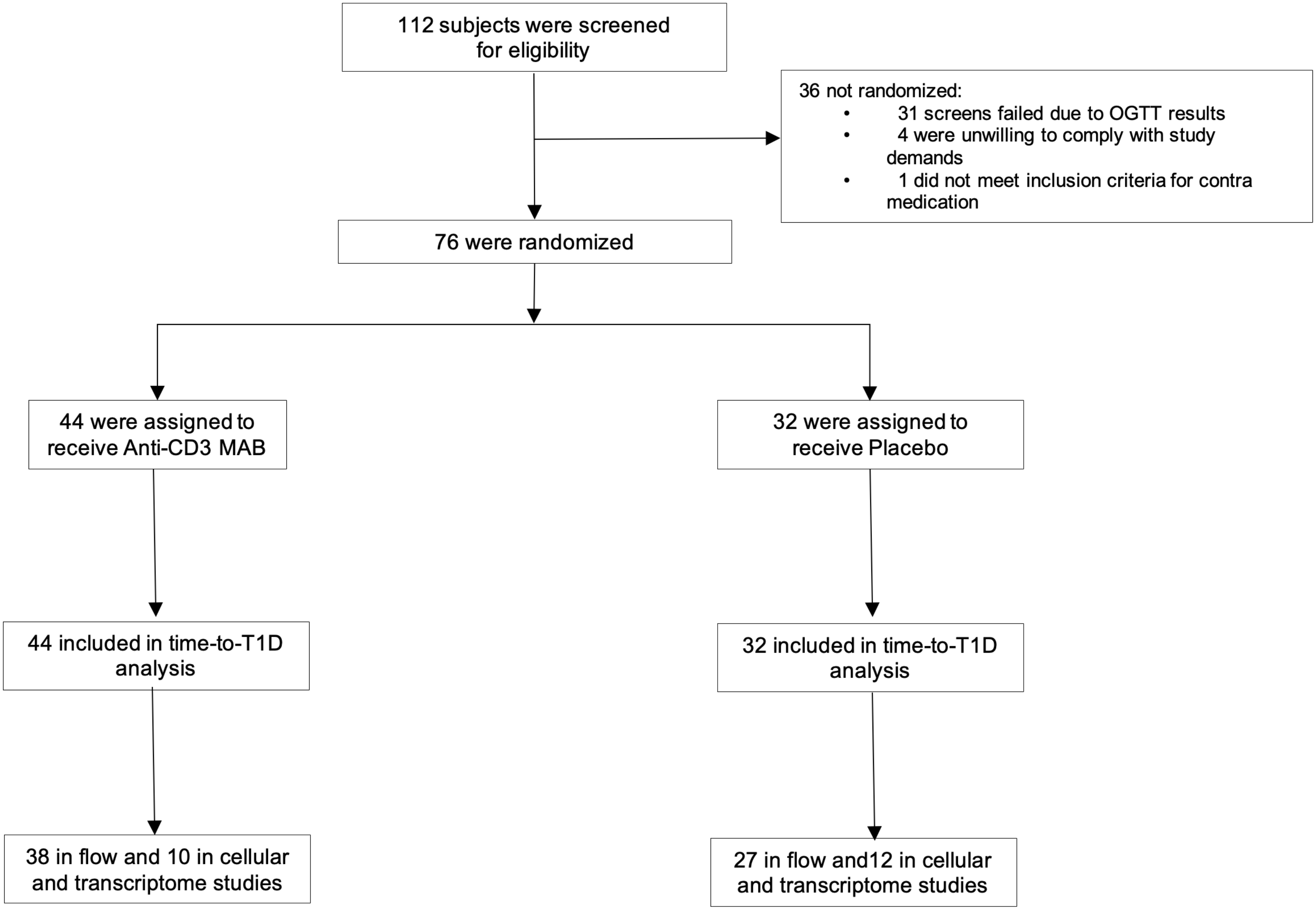

Supp Figure 1. Lledo et al.

Supplemental Figure 1: Consort diagram showing flow of patients through the TN10 study and those included in these analyses

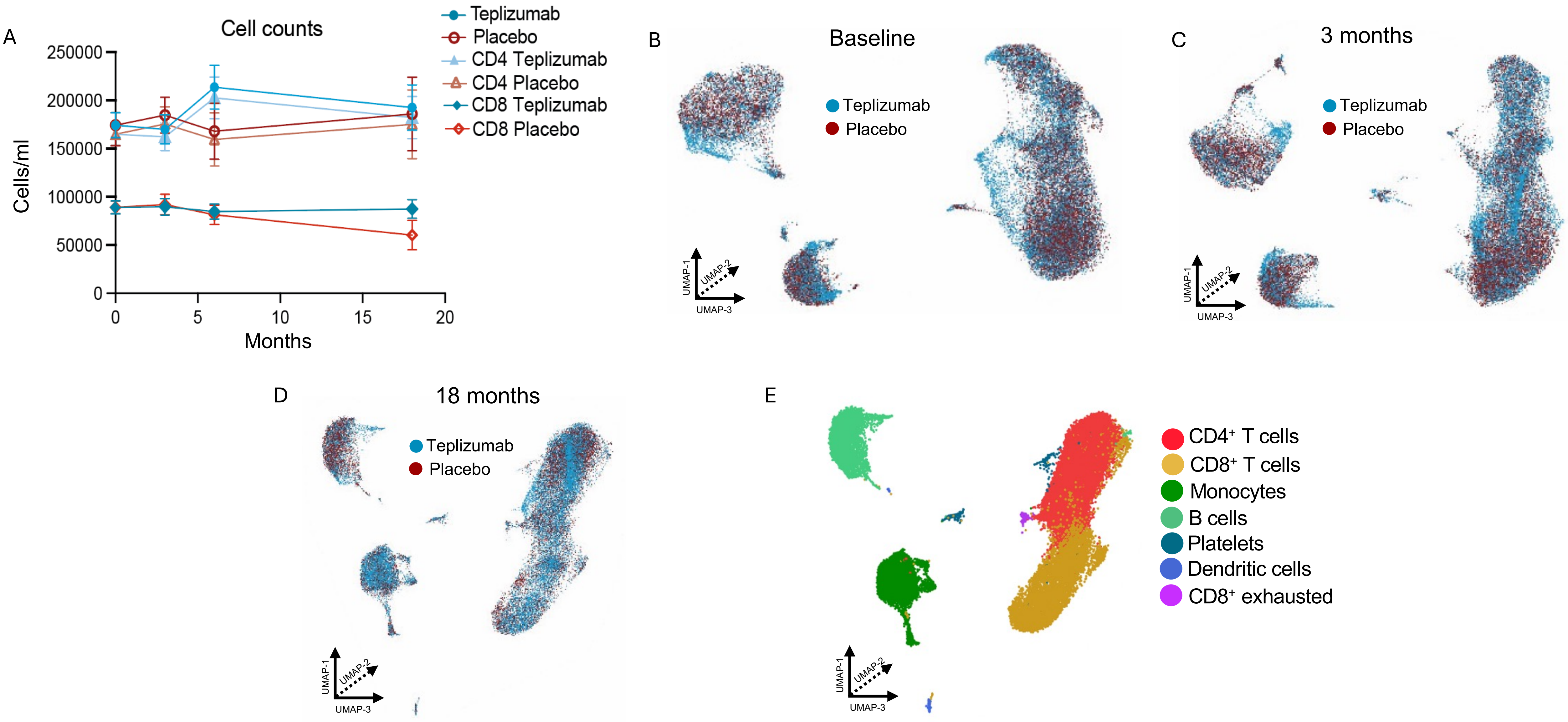

Supp Figure 2. Lledo et al.

Supplemental Figure 2: Cell counts and UMAPs of single cell RNAseq data.

(A) Cell counts by flow cytometry (cells/ml) The mean + SEM from the mixed model are shown.

(B-E) 3D UMAP visualization of cells at baseline (B), 3 months (C) and 18 months (D). Points represent individual cells and color denote treatment arm (B-D) and clusters (E) as labeled (n=7 teplizumab, n=6 placebo).

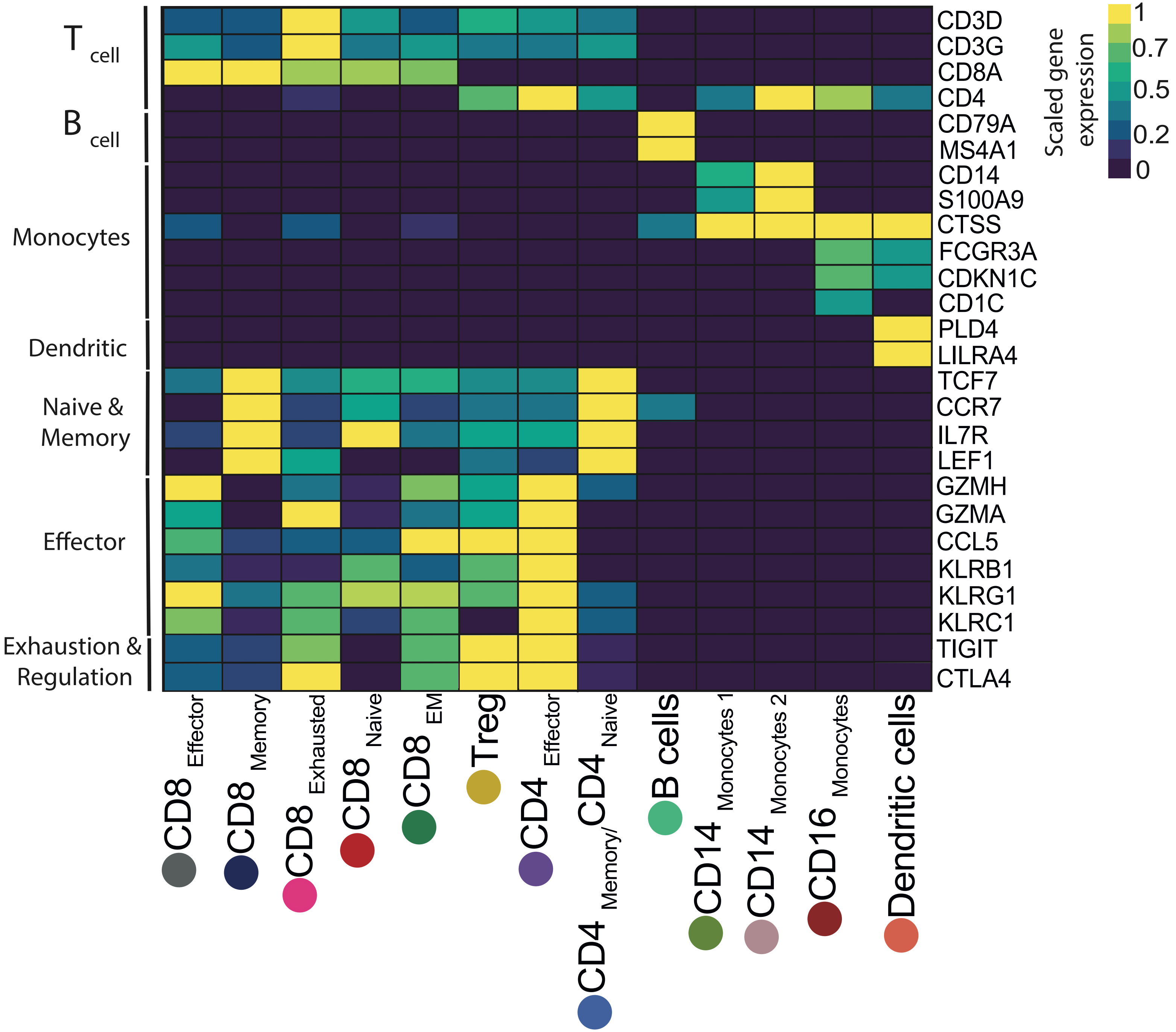

Supp Figure 3. Lledo et al.

Supplemental Figure 3: Heatmap showing gene expression of selected markers used for cluster annotation.

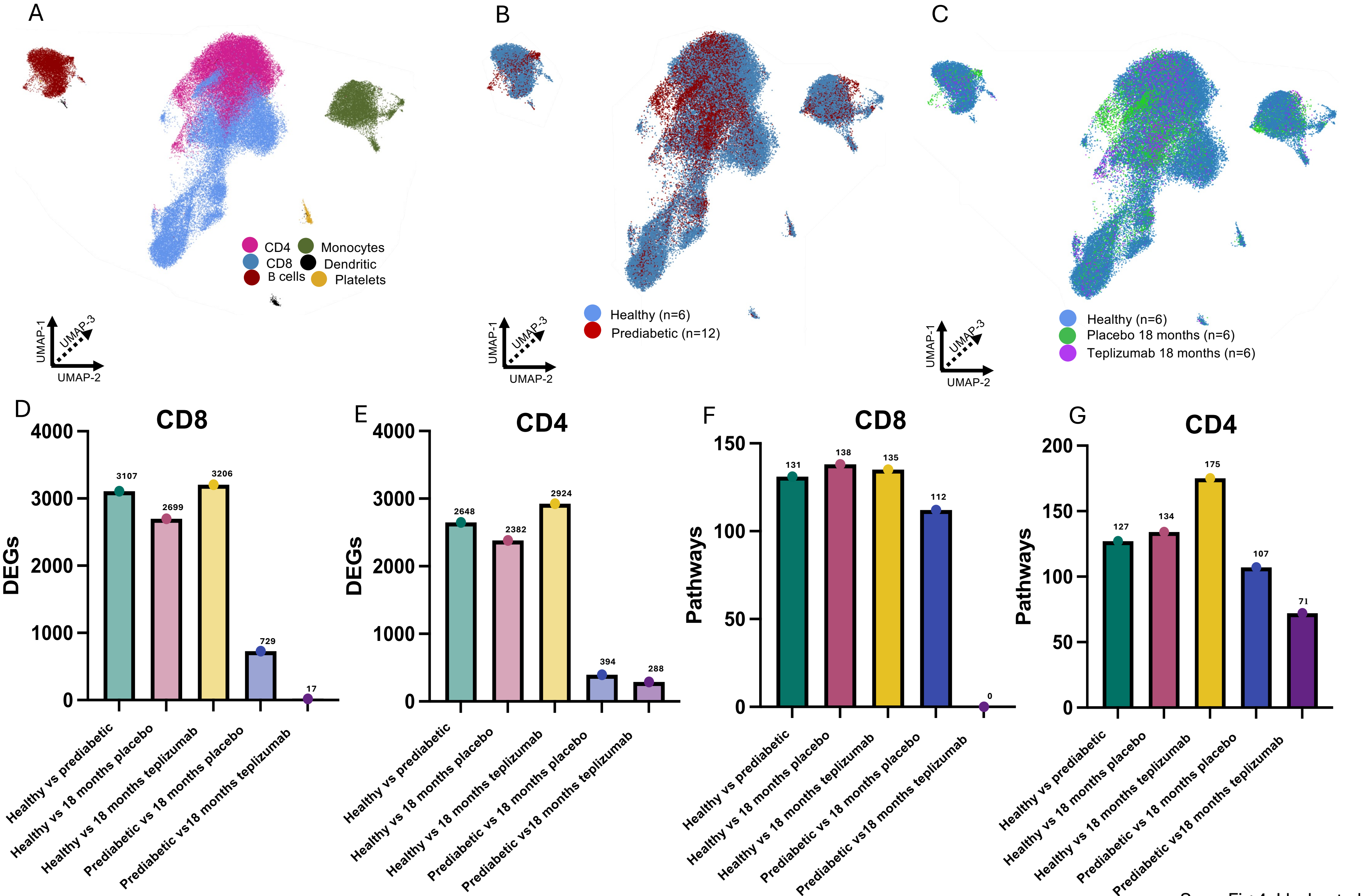

Supp Fig 4. Lledo et al.

Supplemental Figure 4: Comparison of gene expression by single cell RNAseq of samples from TN10 patients and HC (14). (A-C) UMAP visualization of the clusters. (A) Points represent individual cells and color denote cluster classification as labeled. (B) healthy controls (n=6), prediabetic (n=12), (C) healthy controls (n=6) teplizumab 18 months (n=6), placebo 18 months (n=6). (D-G) Bar plots illustrating the number of DEGs (D,E) or IPA (F,G) between healthy controls (n=6) to prediabetic (n=12) or 18 months after placebo (n=6) or teplizumab (n=6) treatment. (D) DEGs in CD8+ T cells in CD8 T cells: healthy vs prediabetic (3107), healthy vs 18 months placebo (2699), healthy vs 18 months teplizumab (3206), prediabetic vs 18 months placebo (729), prediabetic vs 18 months teplizumab (17). (E) DEGs in CD4+ T cells healthy vs prediabetic (2648), healthy vs 18 months placebo (2382), healthy vs 18 months teplizumab (2924), prediabetic vs 18 months placebo (394), prediabetic vs 18 months teplizumab (288). (F) IPAs in CD8 T cells healthy vs prediabetic (131), healthy vs 18 months placebo (138), healthy vs 18 months teplizumab (135), prediabetic vs 18 months teplizumab (112), prediabetic vs 18 months teplizumab (0). (G) IPAs in CD4 T cells: healthy vs prediabetic (127), healthy vs 18 months placebo (134), healthy vs 18 months teplizumab (175), prediabetic vs 18 months placebo (107) prediabetic vs 18 months teplizumab (71).

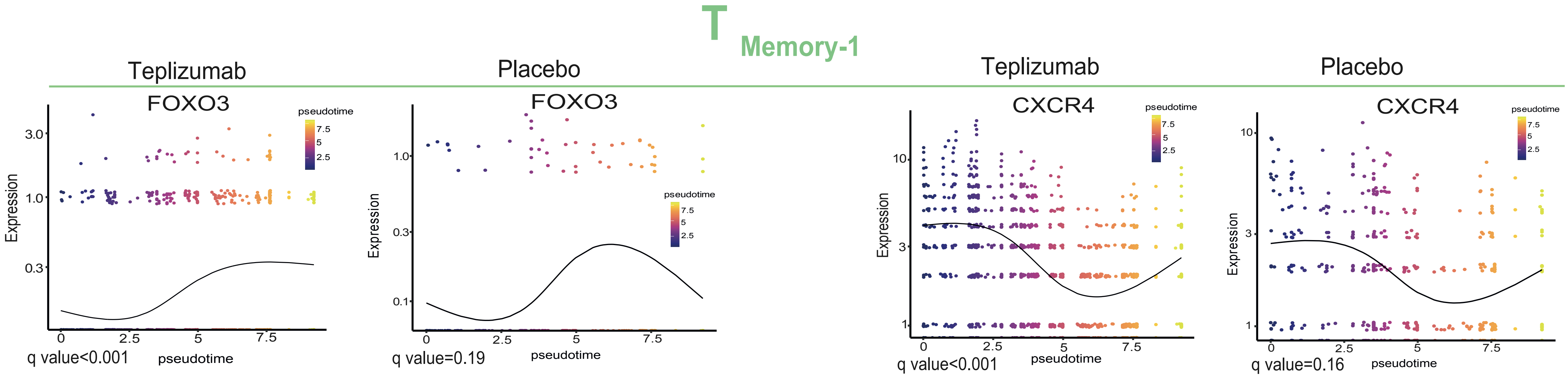

Supp Figure 5. Lledo et al.

Supplemental Figure 5: Gene expression dynamics across the pseudotime in the teplizumab and placebo (groups in the memory-1 branch of differentiation.

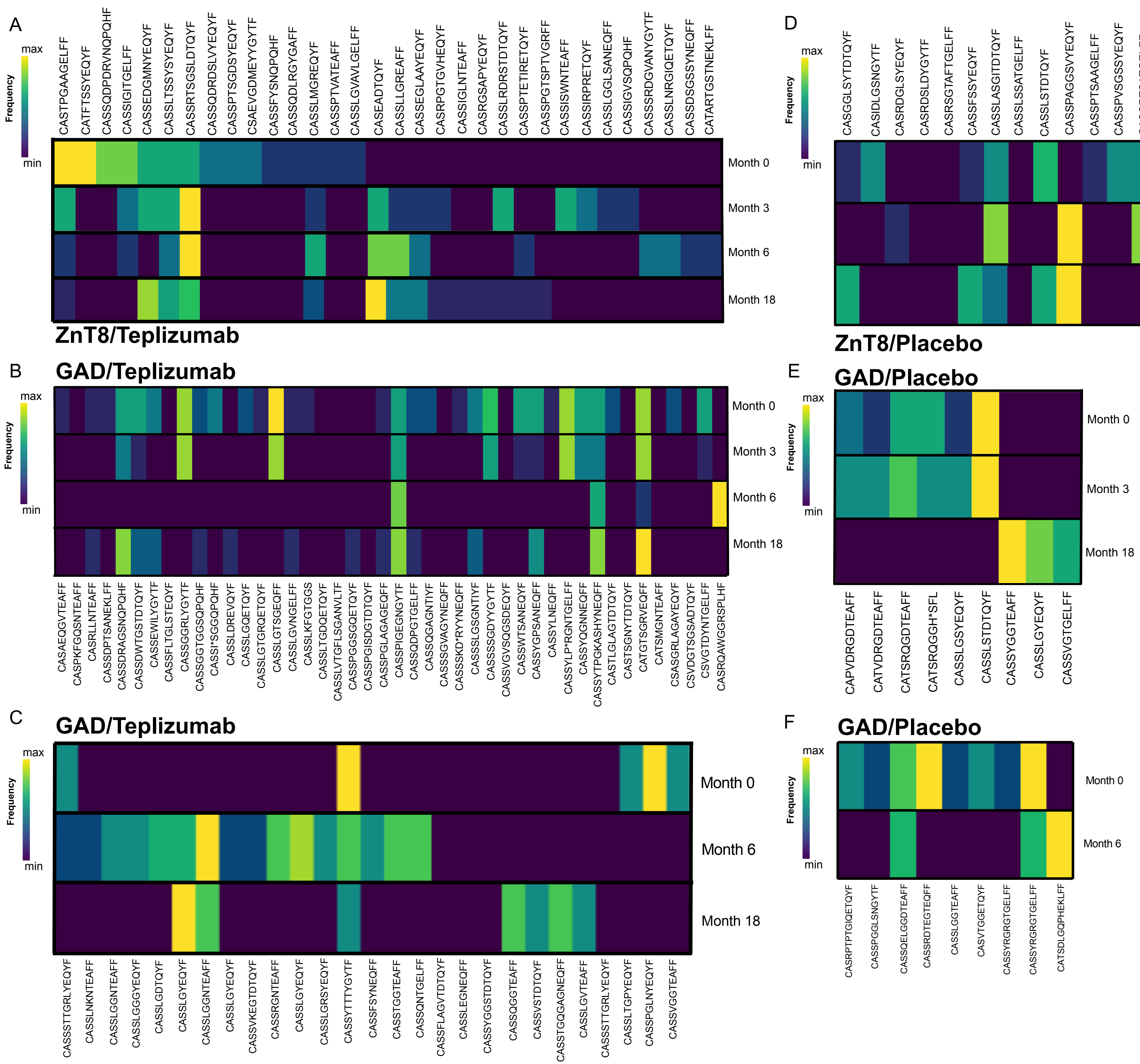

Supp Figure 6. Lledo et al.

Supplemental Figure 6: Heatmaps showing the frequencies of the different Vβ chain sequences of the TCR of the autoantigen reactive CD8+ T cells across the time in representative patients. (A-B-C) teplizumab-treated group (D-E-F) placebo-treated group (n=3). Each row represents a study visit, and each column represents a Vβ chain sequence that was identified in tetramer sorted cells. The heatmaps were constructed using the frequencies of each of the sequences at each study visit.
